# Two Functionally Deviating Type 6 Secretion Systems Occur in the Nitrogen-Fixing Endophyte *Azoarcus olearius* BH72

**DOI:** 10.3389/fmicb.2019.00459

**Published:** 2019-03-12

**Authors:** Xun Jiang, Andreas Beust, Praveen K. Sappa, Uwe Völker, Theresa Dinse, Julia Herglotz, Barbara Reinhold-Hurek

**Affiliations:** ^1^Department of Microbe-Plant Interactions, Faculty of Biology and Chemistry, Center for Biomolecular Interactions Bremen, University of Bremen, Bremen, Germany; ^2^Interfaculty Institute of Genetics and Functional Genomics, University Medicine Greifswald, Greifswald, Germany

**Keywords:** *Azoarcus olearius*, T6SS, proteome, gene expression, motility, endophyte

## Abstract

Type VI protein secretion systems (T6SSs) have been identified in many plant-associated bacteria. However, despite the fact that effector proteins may modulate host responses or interbacterial competition, only a few have been functionally dissected in detail. We dissected the T6SS in *Azoarcus olearius* strain BH72, a nitrogen-fixing model endophyte of grasses. The genome harbors two gene clusters encoding putative T6SSs, *tss-1* and *tss-2*, of which only T6SS-2 shared genetic organization and functional homology with the H1-T6SS of *Pseudomonas aeruginosa*. While *tss-2* genes were constitutively expressed, *tss-1* genes were strongly up-regulated under conditions of nitrogen fixation. A comparative analysis of the wild type and mutants lacking either functional *tss-1* or *tss-2* allowed to differentiate the functions of both secretion systems. Abundance of Hcp in the culture supernatant as an indication for T6SS activity revealed that only T6SS-2 was active, either under aerobic or nitrogen-fixing conditions. Our data show that T6SS-2 but not T6SS-1 is post-translationally regulated by phosphorylation mediated by TagE/TagG (PpkA/PppA), and by the phosphorylation-independent inhibitory protein TagF, similar to published work in *Pseudomonas*. Therefore, T6SS-1 appears to be post-translationally regulated by yet unknown mechanisms. Thus, both T6SS systems appear to perform different functions in *Azoarcus*, one of them specifically adapted to the nitrogen-fixing lifestyle.

## Introduction

To survive and flourish in their natural environment, bacteria have to compete with each other for nutrients and ecological niches. One of the ways bacteria use to gain an advantage over competitors is the Type VI secretion system (T6SS), an one-step protein secretion machinery that allows for the transport of substrates into a targeted organism. Recent research on the T6SS has revealed bacterial killing associated with the T6SS in a variety of organisms, and a growing number of antibacterial effects have been described (Alcoforado Diniz et al., 2015). Also an involvement of the T6SS in the interaction with eukaryotic host organisms has been shown for a variety of bacteria (Hachani et al., 2016).

The T6SS consists of 13 conserved core components termed Tss (type six secretion) and a variable number of accessory proteins termed Tag (type six associated genes) (Silverman et al., 2012; Zoued et al., 2014). The T6SS assembles into four distinct structural complexes: (1) a tube, consisting of hexameric Hcp (TssD) rings with a VgrG (TssI) trimer as a tip that is thought to translocate through the bacterial inner and outer membrane into the targeted organism (Leiman et al., 2009; Basler et al., 2012); (2) A cytoplasmic tube consisting of TssB/C proteins that surrounds the Hcp tube and, via contraction, is thought to provide the energy necessary for the translocation of the Hcp tube. In its contracted state, the tube is disassembled by TssH/ClpV in an ATP dependent process to allow for a reassembly and repeated activation of the T6SS (Bonemann et al., 2009; Kapitein et al., 2013; Lossi et al., 2013); (3) a membrane complex formed by TssJLM that stabilizes the Hcp tube in the membrane is involved in regulating the T6SS activation (Du and Gallon, 1993; Durand et al., 2015); (4) a baseplate that is required for Hcp tube formation and T6SS activation and, as recent results suggest, is consisting of TssE, -F, -G, -K, and VgrG (English et al., 2014; Brunet et al., 2015).

Only very few T6SS have been shown to be post-translationally activated. For the post-translational activation of the T6SS, two independent models have been proposed: a phosphorylation dependent and a phosphorylation independent regulation. The phosphorylation independent regulation has hardly been investigated and was only proposed based on the findings of Silverman et al. (2011). It was shown that a deletion mutant of *tagF* of *Pseudomonas aeruginosa* had elevated levels of Hcp secretion, without the threonine phosphorylation pathway (TPP) being involved as the amount of phosphorylated Fha1 remained unchanged. Furthermore, it was shown that TagF seems to influence the T6SS activity on the level of TssH recruitment.

The more intensively studied post-translational regulatory pathway, however, is threonine-phosphorylation dependent and involves at least three different proteins upstream of TssH (Mougous et al., 2007; Hsu et al., 2009). Upon an environmental cue of yet unknown nature, TagR of *P. aeruginosa* causes homodimerization and autophosphorylation of PpkA (TagE), a serine-threonine kinase that can subsequently bind and phosphorylate Fha1. Fha1 in turn is required for a functional T6SS. Antagonistically to PpkA, PppA (TagG), a serine-threonine phosphatase, controls the phosphorylation state of PpkA and Fha1 (TagH) to ensure a tight regulation of the T6SS activity. Although described exemplary for *P. aeruginosa*, this regulatory system seems to be common to a variety of different organisms.

With recent research on the T6SS focusing on antibacterial killing behavior, it is not surprising that the number of identified antibacterial effector proteins has steadily increased, and more of their functionality has been unraveled (reviewed by Benz and Meinhart, 2014; Durand et al., 2014; Ryu, 2015; Hachani et al., 2016). Identification of these effector proteins has well contributed to our understanding of the T6SS so far, and knowledge of the various types of effector proteins has increased the capacity to predict and identify possible effector protein encoding genes. Still, given the sheer amount of bacteria harboring putative T6SS clusters, many effector proteins are yet to be discovered. Especially effector proteins targeting eukaryotic hosts have hardly been described (Kapitein and Mogk, 2013).

**FIGURE 1:**
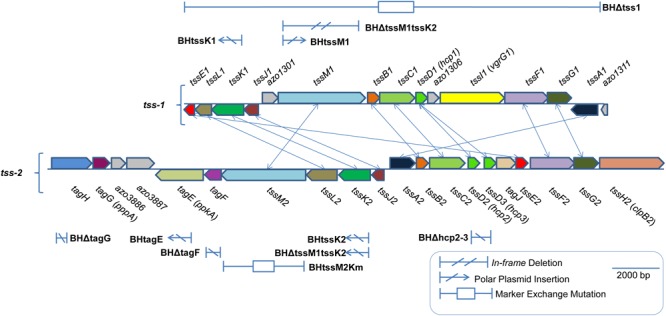
Type VI secretion gene clusters in *Azoarcus olearius* strain BH72. Two gene clusters encoding for proteins of type VI secretion systems, the *tss-1*, and the *tss-2* cluster. Functional homologs connected by lines. Position and type of mutations given. For a mutant carrying mutations in both clusters (BHΔtssM1tssK2), positions in both clusters are shown separately.

*Azoarcus olearius* strain BH72 is a Gram-negative β-proteobacterium that was originally isolated from the roots of Kallar grass [*Leptochloa fusca* (L.) Kunth; Reinhold et al., 1986]. This strain is capable of also infecting rice roots under gnotobiotic conditions, one of the best-studied models for diazotrophic endophyte-plant interactions (Reinhold-Hurek and Hurek, 2011). Its genome was the first endophyte genome to be sequenced (Krause et al., 2006), revealing the presence of two putative T6SS gene clusters, initially termed *imp* and *sci* (further referred to as T6SS-1 and T6SS-2, respectively) (Reinhold-Hurek and Hurek, 2011; Shidore et al., 2012; Figure 1 and Supplementary Table S1). It has further been demonstrated by Hcp secretion that *Azoarcus* sp. BH72 has a functional T6SS, and that a mutation of a T6SS regulatory protein TagG/PppA (Azo3888) positively affected plant colonization efficiency (Shidore et al., 2012). Neither structural components of this system nor secreted effector proteins or implications in interbacterial competition have yet been analyzed for *A. olearius* strain BH72. As other protein secretion systems known from pathogens and symbionts (T3SS and T4SS) are underrepresented in endophytes, while T6SSs are very common (Reinhold-Hurek and Hurek, 2011), T6SSs might play an important role for the life style of endophytes.

We therefore functionally characterized the T6SSs in *Azoarcus* by comparing mutants lacking several putative structural and regulatory components of the T6SS, and by investigating their expression and activation patterns and identify putative effector proteins secreted by them. We could show that, although proteins of both T6SSs of *A. olearius* strain BH72 are expressed under nitrogen fixing conditions, only the T6SS-2 was active. The T6SS-2 activity was post-translationally regulated by two mechanisms, the TPP and by TagF, a homolog of TagF in *P. aeruginosa* that repressed secretion. A secretome analysis confirmed the secretion of Hcp through the T6SS-2 but did not provide proof for secretion of any protein by T6SS-1 under any of the conditions tested. However, gene expression of the *tss-1* gene cluster was strongly induced under conditions of nitrogen fixation, indicating that secretion activity appears to be post-translationally regulated by as yet unknown mechanisms.

## Materials and Methods

### Strains and Growth Conditions

Strains and plasmids are given in Supplementary Table S2. As wild type, *A. olearius* strain BH72 (Reinhold et al., 1986) was used. *Azoarcus* strains were grown aerobically on VM-ethanol medium (Hurek et al., 1995) for growth curves and killing assays, or on SM medium with nitrogen sources (Reinhold et al., 1985; with addition of 0.1 g/l yeast extract and 0.5 g/l NH_4_Cl) for competition and swarming assays. To acquire supernatants of cultures, cells were grown under nitrogen-fixing conditions microaerobically (0.3%) in SM medium without nitrogen sources (Reinhold et al., 1985) in an oxygen-controlled bioreactor (Karg and Reinhold-Hurek, 1996). For conjugation, *Azoarcus* strains were grown aerobically in VM-malate medium (Reinhold et al., 1985; Reinhold-Hurek et al., 1993), using 0.3% peptone instead of beef extract and biotin. *Escherichia coli* strains were grown aerobically on LB agar or in LB medium (Sambrook et al., 1989). If applicable, growth media and agar plates for *E. coli* and *Azoarcus* strains were supplemented with 30 μg/ml of kanamycin.

### Mutant Construction and DNA Manipulations

To generate plasmid integration mutants, a PCR-generated fragment of the 5′-end of the targeted gene was cloned into the vector pK18GGST (Krause et al., 2011) using the restriction sites present on the selected primers (Supplementary Table S3). For *tssM1, tssK1*, or *tssK2*, fragments of 720, 716, or 733 bp length, respectively, were amplified. For *azo3472*, a 638 bp fragment covering 303 bp of the 5′-end of *azo3472* was amplified. The resulting vectors were transferred to *A. olearius* strain BH72 by bi- or tri-parental conjugation, and recombinants were selected based on antibiotic resistance. The correct integration of the plasmids was confirmed by Southern Blot analysis.

To obtain *in-frame* deletion mutants, PCR-generated fragments of the regions upstream and downstream of the targeted gene or region were cloned and then joined into the vector pK18mobsacB allowing sucrose selection (Schäfer et al., 1994), using the restriction sites present on the selected primers (Supplementary Table S3). For BHΔtagF, a 941 bp fragment upstream and an 899 bp fragment downstream of *tagF* were amplified using the *Pfu* DNA polymerase (Thermo Fisher Scientific, Dreieich, Germany) and the primer pairs Up3889For/Rev and Down3889For/Rev, leaving 33 bp at the 5′-end and 99 bp at the 3′-end of *tagF*. For mutant BHΔtagG, a 782 bp fragment upstream and a 983 bp fragment downstream of *tagG/pppA* were amplified using the Pfu DNA polymerase (Thermo Fisher Scientific, Dreieich, Germany) and the primer pairs 3885upF/R and 3885dnF/R, leaving 207 bp at the 5′-end and 84 bp at the 3′-end of *tagG*. For double deletion mutant of hcp2 and hcp3, BHΔhcp2-3, a 1362 bp fragment upstream of azo3897 and a 1249 bp fragment downstream of azo3898 were amplified with primer pairs azo3897F/azo3897R and azo3898F/azo3898R, deleting the entire 1220 bp which contains both hcp2/tssD2 and hcp3/tssD3.

For mutant BHΔIE, a 995 bp fragment upstream of *azo3470* and a 1016 bp fragment downstream of *azo3474* were amplified using the Q5 High-Fidelity DNA Polymerase (New England Biolabs, Frankfurt/Main, Germany) and the primer pairs Up3470For/Rev and Down3470For/Rev, deleting all of the coding sequence of *azo3470*-*74*. For BHΔtssM1tssK2 and BHΔtssM1, an 801 bp fragment upstream and an 823 bp fragment downstream of *tssM1* were amplified using the *Pfu* DNA polymerase and the primer pairs up1302for/rev and downforC/downrev4real, leaving 408 bp at the 5′-end and 532 bp at the 3′-end of *tssM1*. The resulting vectors were transferred to *A. olearius* strain BH72 (or strain BHtssK2 in case of BHΔtssM1tssK2) by conjugation, and single recombinants were selected based on antibiotic resistance. Secondary recombinants were selected based on sucrose selection and the deletion was confirmed via Southern blot analysis. For BHΔtagF, *Not*I (expected fragment size: 3106 bp) and *Eco*RI (expected fragment size: 1747 bp) were used, for BHΔIE, *Aat*I (expected fragment size: 5448) and *Not*I (expected fragment size: 8013) were used, and for BHΔtssM1tssK2, *Eco*RI and *Bam*HI were used (expected fragment size: 800 bp). BHΔfliC3 was constructed previously (Buschart et al., 2012).

In order to perform complementation analysis of *tagF*, a plasmid for *in trans* expression was constructed based on pBBR1MCS-4 (Kovach et al., 1995), named pBBRI-gusA_TagF. The predicted promoter of the *tagF-*containing operon was upstream of *tssJ2* (GTGCCAGCAGTGTGTCGAGATCGAGTTCCAGCGACATGTTG, last G predicted as transcription start point) and was amplified by PCR including the Shine Dalgarno sequence with primers XbaI_Promoter_F and HindIII_promoter_R. The gene *tagF* (*azo3889*) was amplified by using primers HindIII_TagF_F/XhoI_TagF_R including the respective terminal restriction sites. These two fragments were inserted into the pBBRI-gusA vector into *Xba*I/*Xho*I sites. The final construct was confirmed with both restriction enzyme digestion and sequencing. The plasmid was transferred into mutant BHΔtagF by electroporation, yielding BHΔtagF (pBBRI-gusA_TagF). For expression of his-tagged Azo0277, plasmid pBBR1MCS-4-Uni-azo0277 carried a synthetic constitutive promoter, Shine Dalgarno sequence, and gene *azo0277*fused to 6 histidine codons at the 3′-end. In order to generate an *Azoarcus* strain that constitutively expressed *gfp*, a synthetic promoter was linked to a *gfp* gene, and this construct inserted in an intergenic region at the 3′-end of *azo1129* by double homologous recombination. In plasmid pJet1.2-azo1129up+down, the upstream- and downstream fragments of the insertion site were linked by an artificial *Eco*RI restriction site. A gene for enhanced-fluorescence GFP (Egener et al., 1998) was coupled to a synthetic -35/-10 promoter (sequence with *Eco*RI restriction site at 5′-end, listed up to ATG start codon of *gfp*: 5′-GAATTCTTGACAGTTAGCTCAGTCCTAGGTATAATCTCGCGAGCTTAAATTCTGATTGATTGAGGAGAGTTTCAACATG), and cloned into the *Eco*RI restriction site of plasmid pJet1.2-azo1129up+down to yield pJet1.2-azo1129-Pbrick-gfp. For double recombination, the insert was cloned into a mobilizable vector yielding pK18mobsacB-azo1129-Pbrick-gfp.

### Extraction of Proteins

Cellular SDS-soluble proteins were extracted according to Kiredjian et al. (1986). Cells were pelleted at 3200 × g and RT for 10 min and resuspended in 1 ml 1× PBS. Following a 1 min centrifugation step at 16100 × *g* and RT, the cell fresh weight was determined and the pellet resuspended in 1 ml SDS-extraction buffer [62 mM Tris base, 5% (v/v) β-mercaptoethanol, 10% (v/v) Glycerol, 1,8% (w/v) SDS, pH 6.8] per 150 mg fresh weight. The suspension was incubated for 15 min at 95°C, snap cooled on ice for 5 min, followed by separation of cell by centrifugation for 5 min at 16100 × *g* and RT. The protein containing supernatant was transferred and stored at -20°C until further use.

Supernatant proteins were isolated as described before (Shidore et al., 2012). Briefly, cells were removed by centrifugation and supernatant proteins were precipitated in 10% TCA, washed in ethanol and resuspended in sample buffer (8 M urea and 2 M thiourea).

### Western Blot Analysis

Proteins were separated on 12.5% SDS–PAGE gels (Laemmli, 1970) and transferred to a nitrocellulose membrane (Amersham^TM^ Protran^®^, Sigma-Aldrich, Munich, Germany). For detection of Hcp proteins, membranes were blocked in 1× TBS containing 5% skim milk, incubated overnight in 1× TBS with 1% BSA containing 1:3000 rabbit polyclonal antisera against *A. olearius* strain BH72 Hcp proteins (Shidore et al., 2012), followed by a 3 h incubation in 1× TBS supplemented with 1% BSA containing 1:10000 polyclonal swine anti-rabbit immunoglobulins/HRP (Dako, Hamburg, Germany). For detection of NifH proteins, similar conditions were used except for using antiserum against NifH of *R. rubrum*, kindly provided by R. Ludden (Berkeley, CA, United States).

### Detection and Quantification of Swarming Motility

*Azoarcus olearius* strain BH72 and mutant strains grown in SM medium containing nitrogen sources to an OD_578_ of 0.6, were washed twice in the growth medium, and 15 μl of the cultures adjusted to OD_578_ 0.5 were dropped on swarming agar (SM medium without nitrogen sources; with 0.5 g/l malate and 3.5 g/l agar) and incubated for 36 h at 37°C under humid conditions. Pictures were taken and swarming areas calculated relative to the wild type.

### Bacterial Competition and Co-inoculation Assay

*Azoarcus* strains and environmental prey strains were grown in either VM-ethanol medium or SM medium with nitrogen sources, respectively, depending on the medium the competition was performed in. Strains were harvested at an OD_578_ of 1.0–1.2, washed once in the respective growth media and adjusted to an OD_578_ of 1.0. 20 μl of a 1:1 mix of predator and prey were added on top of a VM-ethanol agar plate and incubated for 3 h at 37°C. Alternatively, the mix was added into semisolid SM medium, with or without nitrogen sources and 3.5 g/l agar, and incubated for 12 h at 37°C. For competition under conditions of nitrogen fixation, cells were cultured on N_2_ in air-tight sealed glass tubes (15 ml) on solid SM-N agar (2 ml) with 1.6–1.8% oxygen at 30°C for 24 h. After the incubation, cells were harvested and decadal dilution series plated on VM-ethanol agar. After 3 days of growth at 37°C, colonies were picked, and restreaked on VM-ethanol agar and VM-ethanol agar containing the respective antibiotics to differentiate between the predator and prey strains.

### Beta-Glucuronidase Activity Assay

The beta-glucuronidase activity of transcriptional reporter gene fusion strains was determined as described before (Egener et al., 1999). Briefly, precultures were grown in SM medium with nitrogen source as described above. Main cultures were grown micro-aerobically in either SM medium or SM medium with nitrogen source.

### Cell Disruption, Protein Estimation and Sample Preparation for Mass Spectrometry

The frozen cell pellets were resuspended in 1 ml UT buffer (8 M Urea and 2 M thiourea) and cell disruption was performed by sonication (5 s × 30 s). Afterward, the protein extract was centrifuged for 1 h at 4°C and 16 000 *g*, the resulting supernatant was collected and centrifuged like this one more time. The supernatant containing intracellular protein was stored at -80°C until further use. The protein concentration for the intracellular and extracellular protein fractions (supernatant proteins) was determined using a Bradford assay kit (Pierce, Thermo Fisher Scientific, Bonn, Germany). In order to purify the protein sample from any possible contaminants that might interfere with trypsin digestion, an in-gel trypsin digestion was performed (Kohlstedt et al., 2014) using NuPAGE^®^ Bis-Tris Gels 4–12% (Life Technologies Corporation). Fifteen microgram of protein lysate was loaded on to each well and the separation, staining and destaining was performed according to the manufacture guidelines except for the run time. The run was stopped exactly after 5 min which allowed the sample to be separated by 1 cm. Each lane was then sliced to three pieces which were processed independently. The gel pieces were washed twice with 200 μl of 20 mM NH_4_HCO_3_, 50% (v/v) ACN for 30 min at 37°C and dried by adding 200 μl of ACN two times for 15 min. Trypsin solution (20 ng/μl trypsin in 20 mM ammonium bicarbonate) was added until gel pieces stopped swelling, and digestion was allowed to proceed for 16–18 h at 37°C. Peptides were extracted from gel pieces by incubation in an ultrasonic bath for 30 min in 40 μl of 0.1% (v/v) acetic acid followed by a second extraction with 40 μl of 50% ACN in 0.05% acetic acid. The supernatants containing peptides from the bands corresponding to each lane were collected and pooled and excess ACN was removed by lyophilization. Resultant peptide mixtures were resuspended in 100 μl of buffer A which were then desalted by C18-ZipTip columns (Millipore, Bedford). 100 fMol of alcohol dehydrogenase from yeast (MassPREP ADH Digestion Standard – Waters Corporation, Milford, MA, United States) was added to the desalted peptide mixture as an internal standard. Finally, peptide mixtures were concentrated to 2 μl in a vacuum concentrator and resuspended in 50 μl of buffer A for further LC-MS/MS analysis.

### Shotgun Proteomics (LC-MS/MS) Workflow – Identification and Relative Quantification of Global Proteome

Four hundred nanogram of peptide sample was separated using a nanoACQUITY Ultraperformance LC (Waters Corporation, Milford, MA, United States) equipped with a nano Acquity C18 trap column (2 cm length, 180 μm inner diameter, 5 μm particle size; Waters Corporation) for sample loading and a nano Acquity BEH 130 C18 analytical column (10 cm length, 100 μm inner diameter, 1.7 μm particle size; Waters Corporation) for separation by a gradient with increasing percentage of Buffer B (0.1% (v/v) acetic acid, 5% DMSO in acetonitrile) in Buffer A2 (0.1% (v/v) acetic acid, 2% (v/v) acetonitrile, 2% DMSO in water) at a flow rate of 400 nl/min. In detail, steps of increasing Buffer B percentages were 5% at 1 min, 25% at 65 min, 60% at 80 min, and 99% at 85 min. Separated peptides were analyzed online in data dependent acquisition manner in a LTQ Orbitrap Velos mass spectrometer (Thermo Fisher Scientific Inc., Waltham, MA, United States). Differential analysis of label-free MS data was performed using the Rosetta Elucidator software (Rosetta Biosoftware, Seattle, WA, United States). After aligning the retention time of each MS run, a database (forward and reverse entries of the *Azoarcus* sp. BH72 database, protein entries obtained from ^1^ September 2013) search was performed in Sorcerer version 4.0.4 (Sage-N Research Inc., Milpitas, CA, United States). The search results were filtered for reliable identifications (error < 0.01) which were then used to annotate the peptides. After normalization on feature intensity level and quality filtering, feature intensities were summarized to peptide intensities, and, finally, intensities of unique peptides were summarized to protein intensities. Further data analysis steps were conducted in the Genedata Analyst software package (Genedata, Basel, Switzerland). Protein intensity values were log2-transformed and median normalized, which shifted the median protein intensity of all data sets to the same value. The statistical comparison of sample groups includes four biological replicates from both extracellular and intracellular proteome data obtained from mutant and hypersecretory strains. Ratio values were calculated comparing mutant vs. hypersecretory strain. Proteins were considered as significantly regulated between both groups after a statistical cut off of (*q*-value = 0.05 after Benjamini-Hochberg correction) with a mean fold change of at least 2.

### Targeted LC-MS/MS by SRM (Selected Reaction Monitoring) – Validation of Shotgun Proteomics Data

Relative quantification data for 20 proteins of interest obtained from shotgun proteomics data was validated using a highly sensitive targeted approach via selected reaction monitoring (SRM) technique. Signature peptides for the selected proteins were selected based on the shotgun proteomics data. All peptides included in SRM analysis were required to have 7–25 amino acids in length, be fully tryptic (both N and C termini are formed by cleavage at lysine or arginine). Peptides containing methionine (except for Azo2153 and Azo3474) or cysteine residues were excluded. Peptide uniqueness was confirmed by searching against the protein sequences obtained from *Azoarcus* sp. BH72 database from UniProt. Raw data obtained by targeted SRM were analyzed as described (Zhang et al., 2011; Iuga et al., 2014). Proteins and their corresponding peptides and product ions implemented in SRM experiment are summarized in Supporting Information (SRM_Method.csv).

Peptide separation was carried out using an Acclaim PepMap 100 column (C18, 3 mm, 100 A°, 15 cm bed length, Dionex) and applying a binary gradient from 0 to 40% buffer B (100% acetonitrile [v/v], 0.1% acetic acid [v/v]) in 70 min and to 100% B in additional 15 min at a flow rate of 300 nl min^-1^. Separated peptides were analyzed on a triple quadrupole mass spectrometer (TSQ Vantage, Thermo Fisher Scientific) operated in nano-electrospray mode. For ionization 1600 V of spray voltage and 250°C capillary temperature were used. The resolution for both quadrupoles, i.e., for Q1 and Q3, was set to 0.7 Da (FWHM). The collision gas pressure of Q2 was set at 1.5 mTorr. At least five transitions (product ions) per peptide (precursor) with highly ranked intensities were chosen for targeted analysis. By combining the optimal transitions and the recorded retention time for each target peptide a scheduled method was developed to measure all the peptides of interest in a single MS/MS analysis. The resultant final method included 333 transitions belonging to 57 peptides corresponding to 23 proteins. A TSQ Vantage was operated in SRM (single reaction monitoring) mode and data acquisition was done in scheduled SRM manner with a retention time window of ±4 min for each precursor.

Two technical replicates were analyzed for each sample and for each sample type four independent biological samples were included in the analysis. The raw data obtained were analyzed with the Skyline software version 2.5 (MacLean et al., 2010) to extract peak areas of each peptide. Median peak area from internal standard peptides of alcohol dehydrogenase 1 (ADH_YEAST) was used as a correction factor for the normalization of target peptide peak areas across all MS runs (Zhang et al., 2011; Iuga et al., 2014). The resultant normalized peak areas of each peptide were used to calculate the average peak area of the corresponding proteins. The resultant peak area of protein was used for further statistical validation of the observed changes.

### Bioinformatic Analysis by MultiGeneBlast

In order to compare the gene architecture of gene clusters of *A. olearius* strain BH72 to other organisms, MultiGeneBlast v1.14^2^ was used. For the comparison, an architecture search against a database composed of the GenBank divisions Bacteria, Bacteriophages, and Environmental sequences was performed. 300 Blast hits were mapped per gene at a synteny conservation weight of 1.0 per hit with a minimal sequence coverage of Blast hits of 50% and a minimal identity of 30%. The maximum distance between genes within a locus was set to 20 kbp and 250 loci were returned.

## Results and Discussion

### Elements of Both T6SSs of *Azoarcus olearius* Strain BH72 Are Expressed, With Induction of Gene Expression of *tss-1* Under Conditions of Nitrogen Fixation

**FIGURE 2:**
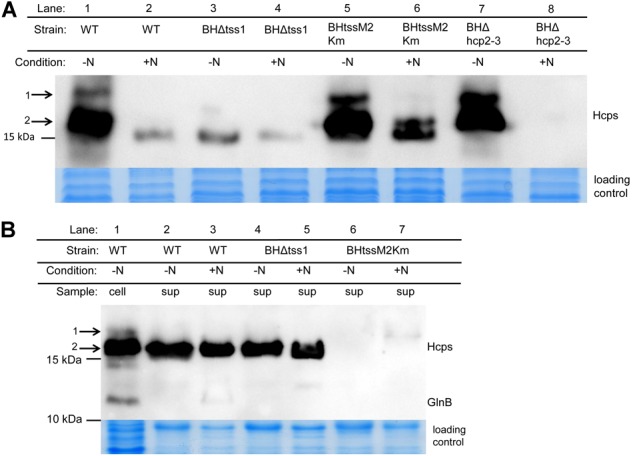
Western Blot analysis for abundance of Hcp in the cellular fraction **(A)** and in culture supernatant **(B)** of *A. olearius* strain BH72 wild type (WT) and three secretion-deficient mutants, BHΔtss1 (TSS-1 inactivated), BHtssM2Km (TSS-2 inactivated), and BHΔhcp2-3 (*hcp2* and *hcp3* of TSS-2 deleted). Hcp was detected with a mixture of three antisera against the three Hcp proteins encoded within the genome of strain BH72. Cultures were grown to an OD_578_ of 0.8 under microaerobic conditions (0.3% oxygen) in SM medium with (+N) or without nitrogen source (–N, conditions of N_2_-fixation). GlnB antibodies were used to test for cellular contaminations of the supernatant fraction. Total proteins were stained with colloidal coomassie to control for equal loading. Protein mass as indicated (in kDa). Hcp Proteins of higher apparent molecular weight (1) and classical molecular weight (2) labeled. Representative figure from at least three independent experiments.

To determine whether the two putative T6SS encoding gene clusters of *A. olearius* strain BH72 are expressed, protein extracts obtained from cells grown under nitrogen fixing conditions or in presence of nitrogen sources were analyzed in a Western blot approach using antibodies directed against Hcp (Figure 2A). A signal was detected at the expected size (17 kDa) and an additional weak signal was observed at 19 kDa in the cellular protein extracts of cells grown under nitrogen fixing conditions (lane 1). Signals were considerably weaker in total cellular protein extracts of cells grown in presence of nitrogen sources (lane 2), and the 19 kDa signal was absent, indicating induction under conditions of nitrogen fixation. In order to differentiate between T6SS-1 and T6SS-2, we created directed mutations in structural genes. The *tss-1* gene cluster was deleted by replacing genes *azo1297*-*azo1311* by a streptomycin/spectinomycin resistance cartridge after double homologous recombination (deletion mutant BHΔtss1). Absence of the 19 kDa signal and decrease of the smaller protein in mutant BHΔtss1 suggested that the majority of the cellular proteins under nitrogen-fixing conditions were encoded in the *tss-1* gene cluster (Figure 2A, lane 3). This was also confirmed by a mutation in a core component of T6SS-2. A marker exchange mutant carrying a kanamycin resistance cartridge in *tssM2*, strain BHtssM2Km, still expressed high levels of Hcp proteins when grown on N_2_ (lane 5). Deletion of both, *hcp2* and *hcp3* genes of T6SS-2 in strain BHΔhcp2-3 allowed a clear differentiation: strong signals were still detectable on N_2_ (lane 7), while under nitrogen surplus, Hcp was not detectable anymore in cells (lane 8). The same was observed for culture supernatants, where Hcp could not be detected in the deletion mutant (Supplementary Figure S1). As the weak 19 kDa protein still appeared in the *hcp2-3* null mutant, it is likely related to Hcp1. Since predicted molecular weights are for Hcp1 17.9, Hcp2 17.6, or Hcp3 17.4 kDa, respectively, Hcp1 might be modified to some extent, yielding a protein of higher apparent molecular weight denoted by 1 in Figure 2 which appeared to be more intense in the Hcp2-3 mutant (lane 7).

With respect to gene expression, transcriptome microarray data indicated induction of the *tss-1*cluster, not the *tss-2* cluster, under conditions of nitrogen fixation (Sarkar and Reinhold-Hurek, 2014). Induction of gene expression was confirmed by using a transcriptional fusion strain BHtssK1, where the fusion of *tssK1* with *gusA* encoding ß-glucuronidase of *Escherichia coli* was integrated into the chromosome. Beta-glucuronidase activity was strongly increased under nitrogen-fixing conditions (845 ± 51 Miller units) in comparison to aerobic growth in the presence of combined nitrogen [SM medium plus nitrogen sources (46 ± 8 Miller units)]. In contrast, a transcriptional *tssK2-gusA* fusion strain in *tss-2* gene cluster (strain BHtssK2) indicated rather constitutive expression of *tss-2* genes under these conditions (393 ± 39 Miller units vs. 485 ± 35 Miller units). Induction of gene expression under conditions of nitrogen fixation, as found here for *tss-1*, was not yet observed for genes encoding Type 6 secretion systems in other bacteria.

### Only the T6SS-2 of *A. olearius* Strain BH72 Is Active in Laboratory Pure Culture

We further investigated the activity of both T6SSs of *Azoarcus* mainly under nitrogen fixing conditions, as genes for both systems were expressed under these conditions. Abundance of Hcp in the culture supernatant was used as an indication for T6SS activity. Total supernatant proteins were extracted from cultures grown in presence or absence of fixed nitrogen sources and subjected to Western blot analysis (Figure 2B). Interestingly, only one signal at 17 kDa was observed in the supernatant samples, irrespective of the growth conditions (lanes 2–5). The *tss-1* null mutant secreted Hcp (lanes 4 and 5) like the wild type, demonstrating that secreted Hcp proteins were encoded in the *tss-2* gene cluster. In contrast, the *tss-2* mutant carrying a mutation in a core component *tssM2* (with unaffected *hcp2, hcp3*) failed to secrete Hcp (lanes 6 and 7). This indicated that, while the *tss-1* gene expression and protein synthesis were induced under nitrogen fixing conditions, the system was not actively secreting, while the major secretion activity was T6SS-2 – mediated. Absence of GlnB as a marker for cellular proteins indicated that presence of Hcp in the supernatant was not caused by leakage of cellular proteins.

### The *A. olearius* BH72 T6SS-2 Shares Homology With the H1-T6SS of *Pseudomonas aeruginosa*

*In silico* studies have indicated that T6SS gene clusters may most commonly be acquired through lateral gene transfer (Boyer et al., 2009), and the ancestry of the gene cluster may give an indication on its function. The *tss-1* cluster of *Azoarcus* belongs to phylogenetic group 4 (Boyer et al., 2009). In order to obtain a better understanding of the putative function of the active T6SS (T6SS-2) of *Azoarcus* sp. BH72, we performed an architecture search using the MultiGeneBlast program (see text footnote^2^). This program uses a given arrangement of protein encoding genes as basis for a BLAST search and assigns a score to different organisms based on the conservation of the overall architecture of the genes (i.e., their arrangement in the genome) as well as a cumulative BLAST score of each individual encoded protein. We observed that the arrangement of genes found in the *tss-2* of *A. olearius* strain BH72 is conserved among many *Pseudomonas* strains and that even the conservation on the protein level is remarkably high (Supplementary Figure S2; average 97% coverage and 54% identity). The H1-T6SS of *P. aeruginosa* has been associated with anti-prokaryotic activity through the use of several effector proteins (Hood et al., 2010; Silverman et al., 2013). While the majority of effector proteins associated with the H1-T6SS are located outside the H1-T6SS gene locus, two effector protein encoding genes (*tse6* and *tse7*) can be found in direct vicinity to it (Sana et al., 2016). However, neither of them are encoded in an operon with the H1-T6SS core components and neither of the effector proteins are conserved in strain BH72. The lack of conserved putative effector proteins associated with the T6SS-2 indicates that it may exhibit a function not associated with anti-prokaryotic activity, however, effector proteins might as well be encoded in other regions.

### Post-translational Regulators of the *Azoarcus* T6SS-2 Act Similar to Their Homologs Involving Phosphorylation in *Pseudomonas aeruginosa*

Post-translation regulation of T6SSs has been studied to great detail in *P. aeruginosa* (PAE). This has led to the discovery of several proteins involved in a TPP that leads to recruitment of the AAA+-type ATPase TssH (ClpV) and subsequent modulation of T6SS activity. While homologs of the proteins involved in the TPP are not conserved among all T6SS containing bacteria, and other forms of post-translational regulation of T6SS activity therefore seem likely, several proteins homologous to proteins of the TPP of *P. aeruginosa* were identified bioinformatically in *A. olearius* strain BH72.

In contrast to the conservation of core components, a variety of post-translational regulatory proteins, while present in both PAE and strain BH72, showed a low degree of conservation: Homologs of Fha1 (PA0077, TagH), PpkA (PA0074, TagE), and PppA (PA0075, TagG) were found in *Azoarcus* (Azo3884, Azo3888, and Azo3885) but showed only on average 46% identity at 51% coverage. This might indicate that the regulation of the T6SS-2 activation could differ from the process found in *P. aeruginosa*.

To investigate whether the putative post-translational regulators encoded in the *tss-2* gene cluster of strain BH72 confer a similar function as their homologs in P. *aeruginosa*, mutants were constructed and the T6SS-2 activity studied via Western blot analysis. In a previous study we have reported that mutation of *tagE/ppkA* led to a loss of secretion (Shidore et al., 2012) as described for *P. aeruginosa* (Mougous et al., 2007). We further constructed an in frame-deletion mutant of *azo3885*, a gene encoding a homolog of the phosphatase *tagG/pppA* of *P. aeruginosa*. Mutation of *tagG* led to a hypersecretory phenotype of *Azoarcus* (Supplementary Figure S3A), indicating that it exhibits a similar influence on T6SS activity as its *P. aeruginosa* homolog (Mougous et al., 2007).

Overall, homologs of all three initially described TPP components of *P. aeruginosa* (TagEGH) are encoded in the genome of strain BH72, and we could show that they convey similar functions as their homologs, probably by threonine phosphorylation dependent mechanism. However, it can be assumed that a different signal is required to activate the TPP, because the *A. olearius* genome does not encode homologs to TagQRST – proteins involved in relaying the environmental signal toward the inner-membrane localized protein TagE/PpkA in *P. aeruginosa* (Casabona et al., 2013).

### Mutation of *tagF* Leads to Increase of the T6SS-2 Activity of *A. olearius* Strain BH72

**FIGURE 3:**
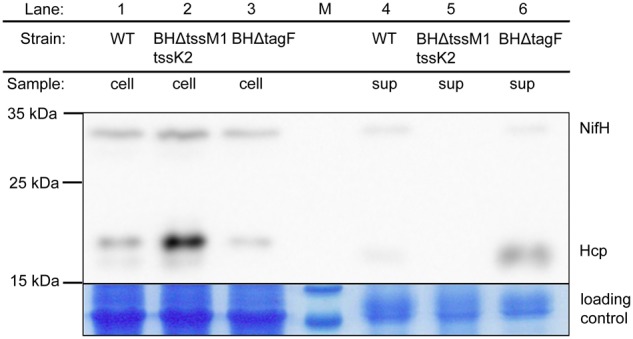
Western Blot analysis for abundance of Hcp in cellular and supernatant fractions of *A. olearius* strain BH72 and the secretion-deficient mutant BHΔtssM1tssK2 (*tss1* and *tss2* inactivated) and regulatory mutant BHΔtagF (*tagF* inactivated). Hcp was detected with a mixture of three antisera against the three Hcp proteins encoded within the genome of strain BH72. Cultures were grown to an OD_578_ of 0.8 under microaerobic conditions (0.3% oxygen) in SM medium without nitrogen source. NifH antiserum was applied to the blot to test for cellular contaminations of the supernatant fraction. Total proteins were stained with colloidal coomassie to control for equal loading. Protein mass as indicated (in kDa). M: size marker. Representative figure from at least three independent experiments.

In addition to the TPP-dependent regulation of the T6SSs of PAE, a phosphorylation-independent repression of T6SS activity by TagF was reported (Silverman et al., 2011). The strain BH72 genome harbors a gene encoding a protein similar to TagF which is located in the *tss-2* gene cluster, *azo3889*. We therefore hypothesized that Azo3889 could have similar functions as TagF. Indeed, for in-frame deletion mutant BHΔtagF, a strong increase in the amount of extracellular Hcp was observed (Figure 3). The absence or low abundance of NifH indicated that this change in phenotype was not caused by an increase of cellular protein contamination or cell lysis, because the iron protein (NifH) of the cytoplasmic nitrogenase complex is a marker for cellular proteins in the supernatant. In order to obtain an isogenic control strain devoid of T6SS-mediated protein secretion, a double mutant in both gene clusters was constructed. Mutant BHΔtssM1tssK2 carried a plasmid insertion inactivating the *tssK2-*operon including *tssM2* and *tagF* of T6SS-2, as well as an *in-frame* deletion of *tssM1* of the *tss-1* cluster (Figure 1). Hcp proteins appeared to accumulate in the cellular fraction, but were absent from the supernatant as anticipated (Figure 3). For complementation analysis of *tagF*, plasmid pBBRI-gusA_TagF was constructed which carried the putative operon promoter for *tagF, tagF*, and a promoterless *gusA* gene for eventual control of expression success by transcriptional fusion. In comparison to the deletion mutant BHΔtagF, the complementation strain BHΔtagF (pBBRI-gusA_TagF) showed decreased secretion of Hcp (Supplementary Figure S3B), as expected for a repressor of T6SS secretion activity.

Thus, TagF of *Azoarcus* was involved in regulating the type VI secretion activity, a process likely to be analogous to the role of TagF in PAE. Proteins sharing homology with TagF can be found in a variety of organisms containing T6SSs (Boyer et al., 2009). In particular, the N-terminal region of TagF appears to be highly conserved (Supplementary Figure S4). Recently, also in *Serratia marcescens* (Ostrowski et al., 2018) and *Agrobacterium tumefaciens* (Lin et al., 2018) the function of TagF was characterized. Together with our results, this indicates a more widespread, general mechanism of TagF as a repressor in regulation of T6SSs activity.

### Whole Secretome Study Confirms T6SS-2 Activity

**FIGURE 4:**
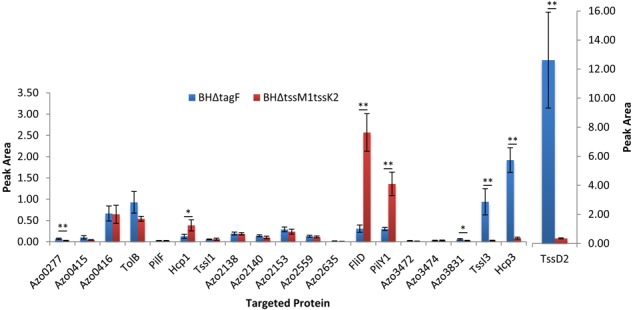
Selected reaction monitoring (SRM) results from 20 candidate proteins in the culture supernatant of the hypersecreting mutant (BHΔtagF) and mutant BHΔtssM1tssK2 (*tss1* and *tss2* inactivated) of *A. olearius* BH72. A detailed description of the candidate proteins can be found in Supplementary Table S5. Averages of four independent biological replicates depicted. Significant differences as indicated (^∗^*p* < 0.05; ^∗∗^*p* < 0.01).

To confirm the effect of the mutation of *tagF* on the T6SS-2 activity, we analyzed the secretome composition of strain BHΔtagF under conditions of nitrogen fixation. In an effort to identify proteins secreted through the T6SSs of *Azoarcus* sp. BH72, the secretome composition of BHΔtagF was compared to the isogenic strain BHΔtssM1tssK2 deficient in T6SS-1 and T6SS2-mediated secretion. Total cellular and supernatant proteins were extracted in four replicates from a T6SS-deficient (BHΔtssM1tssK2) and the hypersecreting (BHΔtagF) T6SS mutant and used for total protein analysis via shotgun proteomics (LC-MS/MS) (Supplementary Tables S4–S6 and Figure S5). The analysis confirmed that the T6SS-2 was active, with Hcp2, Hcp3, and TssI3 (VgrG3) being 48.7-, 23.9-, and 96.1-fold more abundant in the hypersecreting mutant strain (Supplementary Table S5, raw data in Supplementary Table S6). Their hypersecretion was further confirmed by a selective reaction monitoring (SRM) approach (Figure 4 and Supplementary Table S7). As VgrG3 appears downregulated together with Hcp2 and Hcp3 in the *tss-2* cluster mutant, VgrG3 might be the spike used to fire the T6SS-2 system. VgrG and Hcp proteins have frequently been identified in T6SS-dependent proteomes (Altindis et al., 2015; Eshraghi et al., 2016).

In a number of bacteria, mass spectrometry-based proteomic approaches have identified effector proteins (Ho et al., 2014; Altindis et al., 2015; Eshraghi et al., 2016). The secretome comparison of the two T6SS mutants resulted also in the identification of several proteins displaying an altered abundance (Supplementary Figure S5, Figure 4, and Supplementary Table S5). A preliminary bioinformatic analysis suggested that the majority of identified proteins was not related to T6SS functionality and did not conform to the expected parameters of a putative effector protein (absence of a signal sequence, absence of domains implicating the protein in known functions and absence of homologs with known non-T6SS related function in other organisms). Abundant stable proteins are frequently observed in extracellular proteomes (Eshraghi et al., 2016), while removal of outer membrane vesicles may help in reducing contaminations by outer membrane and periplasmic proteins (Altindis et al., 2015). Azo0277, however, was only identified in the supernatant of the hypersecretion mutant BHΔtagF (Supplementary Table S5), and did not contain any known domains, signal peptides or homologs with known function in other organisms; therefore, it might be a potential effector protein. Due to the absence of any functional domains or homologs in other organisms, no functional predictions can be made for this protein. The T6SS-dependent secretion of Azo0277 was confirmed by an SRM approach (Figure 4). To further corroborate its secretion, an *Azoarcus* strain expressing an C-terminally His-tagged version from plasmid pBBR1MCS-4-Uni-azo0277 was constructed. In the wild type background, Azo0277His was detected by Western blot analysis in the cellular fraction, but not in the supernatant. However, the TagF mutant in which T6SS-2 was more active, a weak secretion of Azo0277His could be observed (Supplementary Figure S6), suggesting a T6SS-2 dependent mechanism. Whether this putative effector is capable of harming other bacteria is not known, however, cytoplasmic expression in *E. coli* (Supplementary Figure S6) did not lead to death or growth inhibition.

### Antibacterial Activity and a Putative T6SS Effector Protein of *Azoarcus olearius*

Often T6SS-secreted effector proteins are toxins mediating competitive advantages over other bacterial strains. In an attempt to unravel whether strain BH72 generally shows antibacterial activity, competition (“killing”) assays were performed in which the wildtype was competing with related environmental strains (*Azovibrio restrictus* S5B2^T^ and *Azoarcus tolulyticus* Td-1^T^) that lack presence of a T6SS according to genome analysis, and therefore most likely also lack relevant immunity proteins. A putative antibacterial killing was investigated under several conditions, including co-incubation on solid agar plates and in semi-solid SM agar with or without nitrogen sources, in an attempt to stimulate secretion of putative effector proteins. However, no reduction of cell number could be detected for the strains tested (example for nitrogen-fixation conditions in Supplementary Figure S7, where *Azoarcus* wild type and T6SS-null mutants dominate due to better growth rather than killing). As *Escherichia coli* is often a suitable prey, it was also subjected to competition experiments. These were also carried out under conditions of nitrogen fixation. The tagged wild type strain BHgfp or the T6SS secretion deficient strain BHΔtssM1tssK2 did not induce decrease or increase, respectively, in abundance of *E. coli* in co-culture; high numbers of *Azoarcus* were due to better growth. Even the T6SS-2 hypersecreting strain BHΔtagF did not inactivate *E. coli* (Supplementary Figure S8). This indicated that antibacterial activity was not apparent for the constitutive T6SS-2 system. However, as T6SS-1 activity was not detectable under laboratory conditions, antibacterial effectors might not be secreted under these conditions.

However, known effector proteins may occur in *Azoarcus.* Russell et al. (2013) identified an effector/immunity pair in a heuristic approach that displayed similarities to predicted proteins in *A. olearius* BH72. Specifically, the study proposed that the gene *azo3471* encodes for a putative Tle (Type VI lipase effector) protein similar to Tle-3, while *azo3472*-*azo3474* encode for the required putative immunity proteins. The position of these genes in direct vicinity of a VgrG homolog-encoding gene (*azo3470, vgrG2*, and *tssI2*) made this assumption even more likely, as effector/immunity pairs are mostly found encoded next to a *vgrG* homolog (Barret et al., 2011). To test this hypothesis, we sought to create a mutant strain in which expression of the immunity protein encoding genes (*azo3472*-*azo3474*) were disrupted (BHazo3472). If Azo3471 conveyed anti-bacterial properties, we would expect BHazo3472 to not be viable. However, we were able to successfully create the mutant strain. To exclude that incorrect localization of the effector protein prevents it from exhibiting a lethal effect, we further tested whether a wildtype strain would be able to reduce the mutant strains viability upon co-incubation. However, despite several conditions in which these strains were co-incubated, no reduction in viability of BHazo3472 was observed (data not shown). The secretome study revealed that neither VgrG2 (TssI2, Azo3470) nor Azo3471 or Azo3472-3474 were detected to be secreted through the T6SS-2 of strain BH72 (Supplementary Table S5 and Figure 4). In addition, a whole genome microarray analysis comparing nitrogen fixing and non-nitrogen fixing conditions revealed that the gene cluster surrounding *azo3471* (*azo3470*-*azo3474*) was induced under nitrogen fixing conditions, similar to the *tss-1* gene cluster (Sarkar and Reinhold-Hurek, 2014). These findings could hint toward Azo3471 being secreted through the T6SS-1 and that it might exhibit yet unknown functions related to the hypothesis that the T6SS-1 is involved in microbe-plant interaction.

### Both T6SSs of *A. olearius* Strain BH72 Are Involved in Regulation of Cell Motility

**FIGURE 5:**
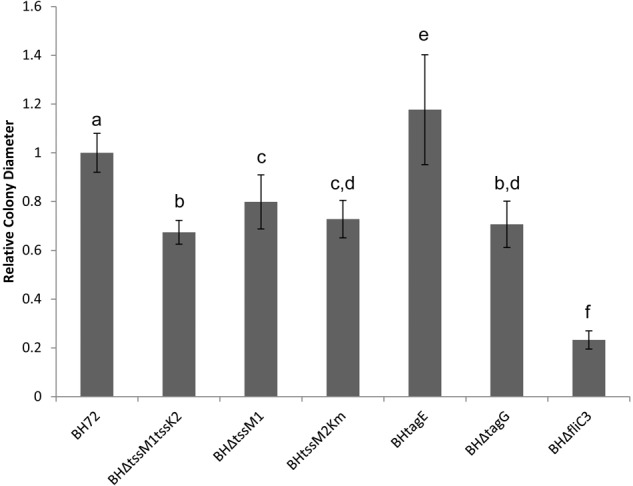
Analysis of swarm colony size of *A. olearius* strain BH72 and four mutants: BHΔtssM1tssK2 (*tss1* and *tss2* inactivated), BHΔtssM1 (*tss1* mutant), BHtssM2Km (*tss2* mutant), and BHΔflic3 (flagellin mutant). Colony diameter was measured after 36 h of incubation on swarming agar and calculated relative to the swarming diameter of the wild type (strain BH72). Statistically significant differences in colony diameter, determined by Welch’s *t*-test, are indicated by different letters (*p* < 0.05). Data from three independent experiments.

Comparing the differently abundant proteins in the supernatant of the two mutants, an overrepresentation of motility proteins in the supernatant of the T6SS – negative mutant was apparent. A total of eight flagella-associated proteins (2.1- to 6.2-fold), one type IV pilus associated protein (2.9-fold) and four chemotaxis-related proteins (8.4- to 9.9-fold) were found more abundantly in the BHΔtssM1tssK2 mutant supernatant (Supplementary Table S5), indicating a close connection between the activity of the T6SS and the export of motility related proteins. These results are surprising as previous reports in other systems have indicated that mutation of *tssM* leads to a decrease in motility in avian pathogenic *E. coli, P. aeruginosa*, and *Vibrio cholera* (Das et al., 2002; de Pace et al., 2011; Lin et al., 2015). Because of this putative contradiction, we investigated a possible correlation between T6SS genes in strain BH72 and its motility. Three different mutants in both or single T6SS gene clusters were analyzed, and each mutant (BHΔtssM1tssK2, BHΔtssM1, and BHtssM2Km) showed a reduction in swarm colony size of approximately 20–30% in comparison to the wild type (Figure 5). As a negative control, strain BHΔfliC3, a non-motile flagellin mutant known to have drastically reduced swarming capacity (Buschart et al., 2012), showed a reduction of about 77% (Figure 5). These results support previous findings that mutation of *tssM* leads to a decrease in motility (Das et al., 2002; de Pace et al., 2011; Lin et al., 2015). Interestingly, in contrast to the findings of Decoin et al. (2015), mutations in either T6SS of strain BH72 led to a decrease in motility, while mutation of both systems did not cause a cumulative stronger reduction in motility; this hinted toward a common regulatory pathway being affected by both mutations. Analysis of the swarming capacity of mutants of post-translational regulators of the T6SS-2, BHtagE (Shidore et al., 2012) and BHΔtagG (Figure 5), indicated that not the loss of T6SS activity but rather a secondary function of these proteins was related to changes in motility of the bacterium.

## Conclusion

**FIGURE 6:**
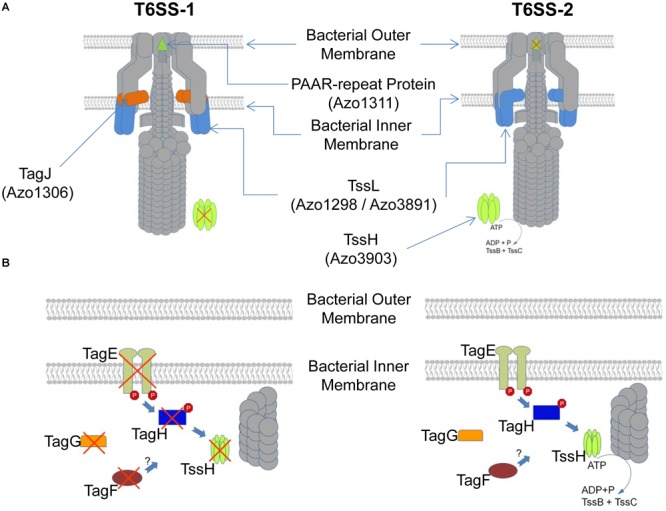
Schematic depiction of differences in the T6SS-1 and T6SS-2 of *A. olearius* BH72 based on presence or absence of the respective protein encoding genes in either T6SS gene cluster. **(A)** Depiction of the T6SS apparatus. Components encoded in both gene clusters depicted in gray, a red cross indicating absence of the component. **(B)** Depiction of the expected post-translational regulatory cascade of each apparatus. Components encoded in both gene clusters depicted in gray, a red cross indicating absence of the component.

Type VI secretion systems have been identified in plant-associated bacteria, but in few systems their functionality has been dissected (Bladergroen et al., 2003; Ryu et al., 2006; Zhang et al., 2014). Here we could show that the nitrogen-fixing endophyte *A. olearius* strain BH72 possesses two T6SS encoding gene clusters, and elements of both secretion systems are expressed and translated. However, both systems deviate functionally (Figure 6). On the post-translational level, several components of the threonine-phosphorylation pathway-dependent and -independent regulatory pathways are encoded in the *tss-2* gene cluster but absent in the *tss-1* gene cluster, including the terminal receptor of these pathways, TssH. Functional analysis of these components revealed two distinct post-translational regulatory mechanisms for T6SS-2, similar to the ones described for *P. aeruginosa*. However, they did not affect T6SS-1 activity, indicating lack of regulatory cross-talk between the two secretion systems. Comparison of the two *tss* gene clusters with those of various other organisms revealed a conserved synteny of the T6SS-2 resembling the one found in several *P. aeruginosa* strains. In contrast, comparably less conservation was observed for the T6SS-1, which groups into T6SS subgroup IV where several plant-associated bacteria cluster (Boyer et al., 2009). Overall, these findings seem to indicate that the T6SS-2 functionality might be rather conserved while the T6SS-1 functionality might be more specific to the lifestyle of *A. olearius* BH72.

This assumption is supported by an unusual gene expression pattern: expression of the *tss-1* gene cluster is strongly induced under conditions of nitrogen fixation. This is particularly intriguing because conditions of nitrogen fixation appear to be typically coupled to the endophytic lifestyle of strain BH72 (Egener et al., 1999; Hurek et al., 2002; Sarkar and Reinhold-Hurek, 2014). Surprisingly, although cellular protein expression of the T6SS-1 was observed under nitrogen fixing conditions, no T6SS-1 activity could be detected. None-functionality of the T6SS-1 due to absence of a *tssH* gene cannot be excluded, though T6SS activity was observed in other organisms lacking *tssH* homologs (Bröms et al., 2012). Moreover, the T6SS-1 could be recruiting *clpV* (*tssH*) homologs encoded outside the *tss-1* gene cluster of strain BH72, e.g., *azo1566* (*clpB*) or *azo3903* (*clpB* of the *tss-2* gene cluster). Most T6SSs studied so far displayed a basic activity when expressed, as indicated by Hcp proteins found in the supernatant of various strains (Pieper et al., 2009; Silverman et al., 2011; Wu et al., 2012; Salomon et al., 2014). In the majority of tested bacteria, a mutational induction of *tss* gene expression was accompanied by an increase in secretion (Schell et al., 2007; Records and Gross, 2010). Thus, the T6SS-1 studied here appears to be post-translationally regulated by a yet unknown mechanism, and conditions for secretion activation still need to be discovered.

## Author Contributions

BR-H, AB, and XJ planned the experiments. PS, UV, and AB carried out mass spectrometry-based proteomic analysis and performed data analysis. JH carried out the transcriptional fusion experiments. AB, TD, and XJ constructed all other mutants and carried out Western Blot experiments. BR-H, AB, and UV wrote the manuscript.

## Conflict of Interest Statement

The authors declare that the research was conducted in the absence of any commercial or financial relationships that could be construed as a potential conflict of interest.

## Footnotes

1 www.uniprot.org

2 http://multigeneblast.sourceforge.net
