# Supplementary material for: Two Functionally Deviating Type 6 Secretion Systems Occur in the Nitrogen-Fixing Endophyte Azoarcus olearius BH72
Source: Front Microbiol. 2019 Mar 12;10:459. doi: 10.3389/fmicb.2019.00459 (PMC6423157; doi:10.3389/fmicb.2019.00459)
Supplement: Supplementary file 3 [file Data_Sheet_1.PDF]

Jiang et al., Supplementary Figures and Tables

**Lane**	**1**	**2**	**3**	**4**	**5**	**6**
Condition:	-N	-N	+N	-N	-N	+N
Strain:	WT	BHΔhcp2-3	BHΔhcp2-3	WT	BHΔhcp2-3	BHΔhcp2-3
Sample:	cellular	cellular	cellular	supernatant	supernatant	supernatant

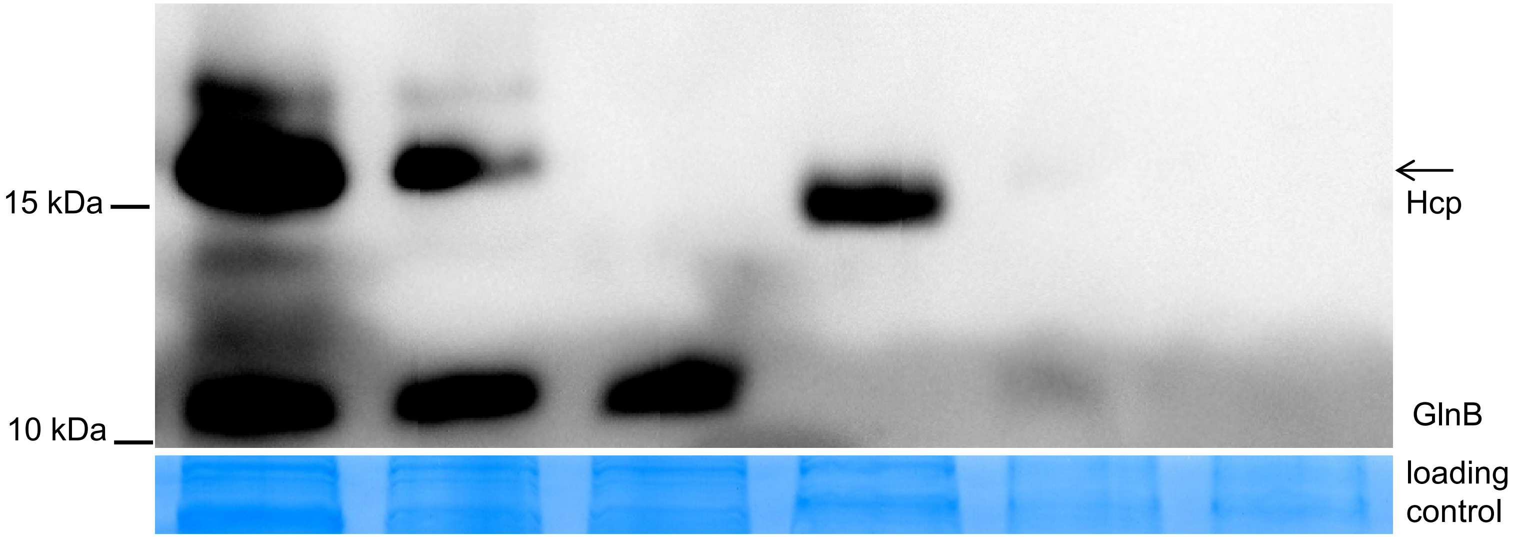

Supplementary Figure S1 │ Western Blot analysis for abundance of Hcp in cellular and supernatant fractions of *Azoarcus olearius* BH72 and a deletion mutant of the *hcp* genes in the *tss2*-cluster BHΔhcp2-3. Hcp was detected with a mixture of three antisera against the three Hcp proteins encoded within the genome of strain BH72. Cultures were grown under N_2-fixing conditions under microaerosis in SM medium without nitrogen source (- N) or under aerobic conditions in SM medium with nitrogen source(+ N) to an OD_{578} of 0.7-0.8. GlnB antiserum were used to test for cellular contaminations of the supernatant fraction. Total proteins were stained with colloidal coomassie to control for equal loading. Protein mass as indicated (in kDa). M: Size marker.

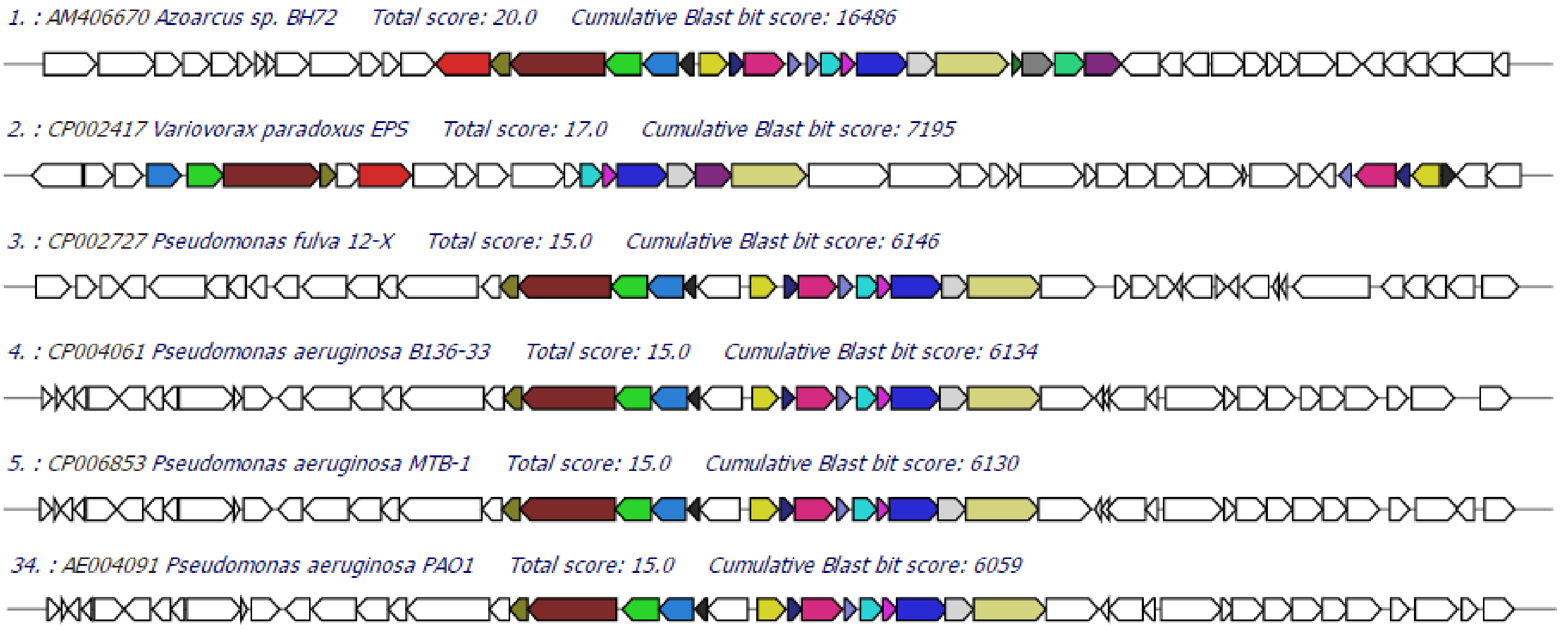

Supplementary Figure S2 | MultiGeneBLAST comparison of the T6SS-2 architecture of *Azoarcus olearius* strain BH72. Results were obtained from the GenBank divisions Bacteria, Bacteriophages and Environmental sequences. A synteny conservation weight of 1.0 was used and the minimal coverage and identity of BLAST hits was set to 50 % and 30 %, respectively. 5 highest scoring hits and the results for *Pseudomonas aeruginosa* PAO1 are depicted.

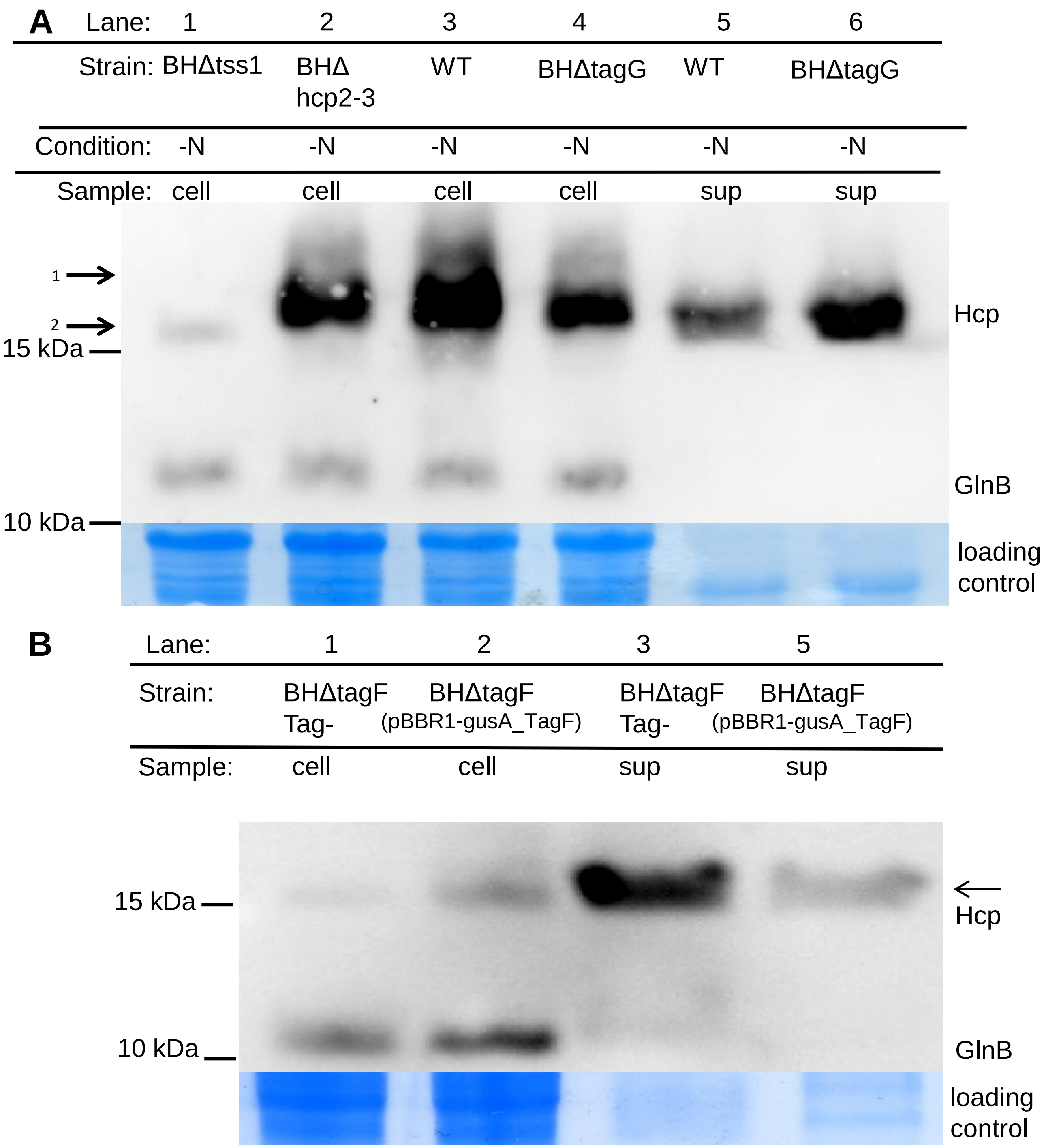

Supplementary Figure S3 │ Western Blot analysis for abundance of Hcp in cellular and supernatant fractions of *Azoarcus olearius* strain BH72 and the hyper-secretion mutant BHΔtagG (A) or BHΔtagF. Control strains were wild type (WT), a deletion mutant of the *tss1*-gene cluster BHΔtss1, and a deletion mutant of the *hcp* genes in the *tss2*-cluster BHΔhcp2-3 (A). Hypersecreting mutant BHΔtagF and complementation by *tagF* in *trans* in mutant BHΔtagF (pBBR1-gusA_TagF). Hcp was detected with a mixture of three antisera against the three Hcp proteins encoded within the genome of strain BH72. Cultures were grown (A) under N_2-fixing conditions to an OD_{578} of 0.7-0.8 under microaerosis in SM medium without nitrogen source, or (B) to an OD_{578} of 0.3-0.4 under aerobic conditions in SM medium with nitrogen source. GlnB antiserum were used to test for cellular contaminations of the supernatant fraction. Total proteins were stained with colloidal coomassie to control for equal loading. Protein mass as indicated (in kDa). M: Size marker. [1]: Hcp-1. [2]: Hcp-1/-2/-3.

A)

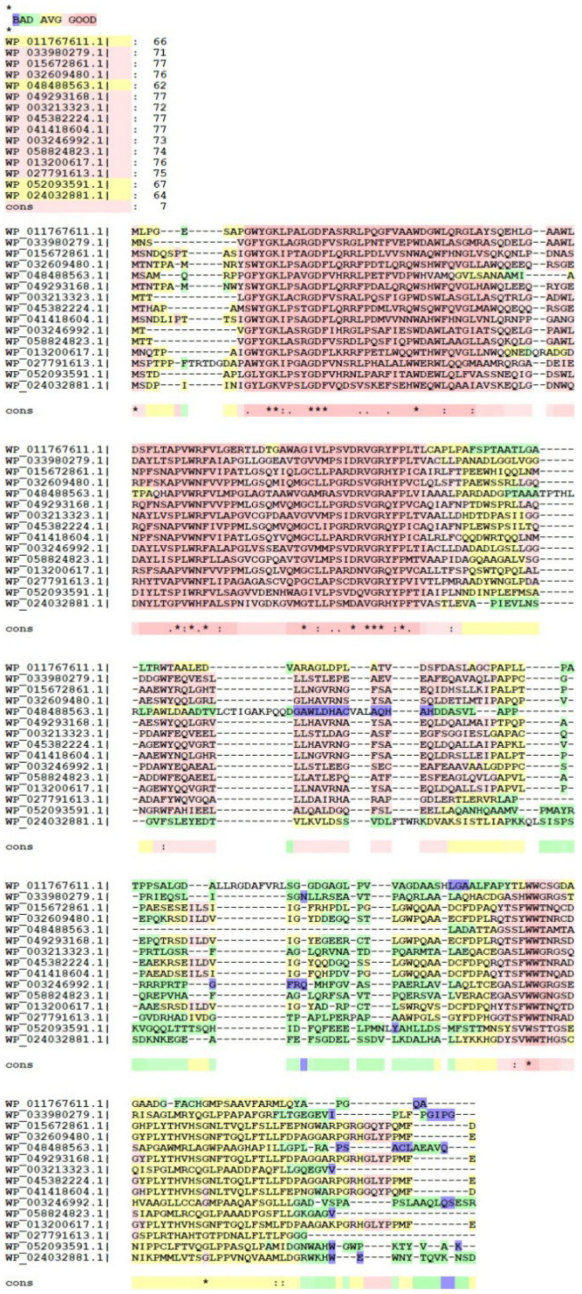

B)

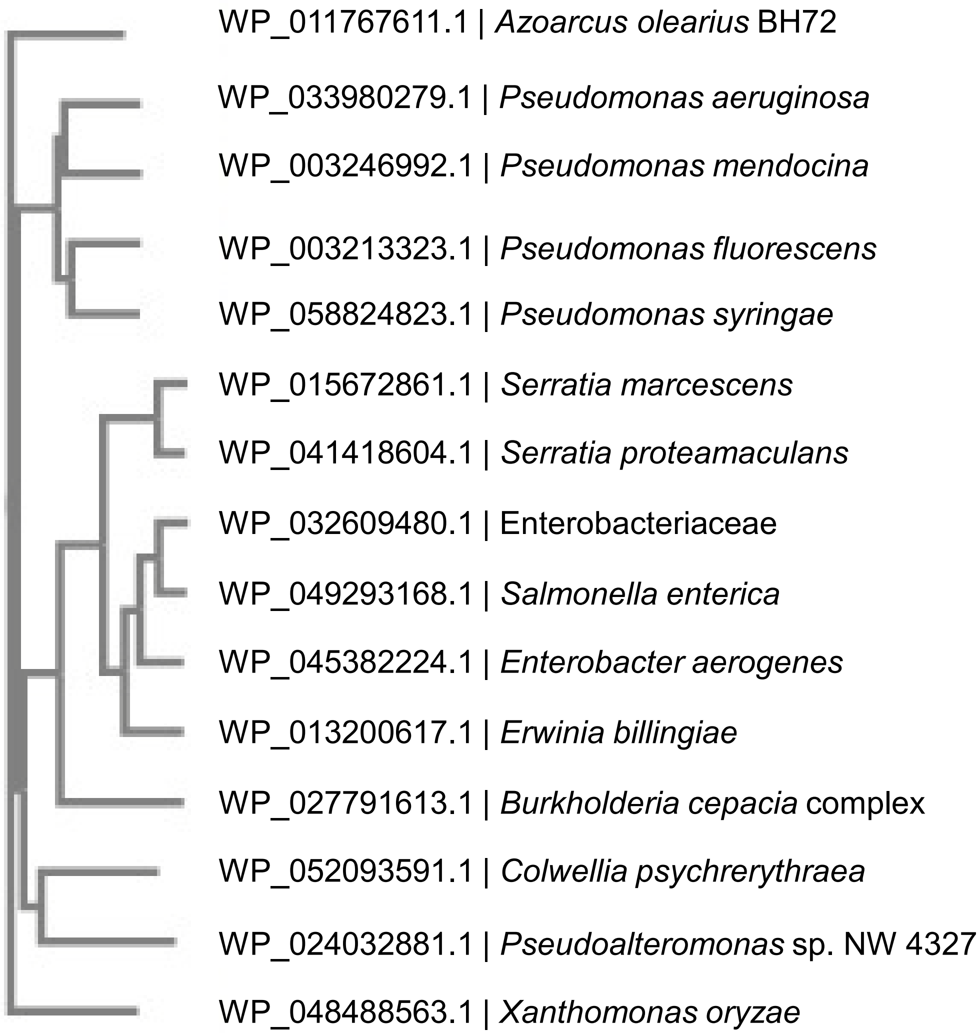

Supplementary Figure S4 │ Multiple sequence alignment and phylogeny of different TagF homologues found in several species. (**A**) Multiple Alignment of *Azoarcus* TagFand 14 TagF homologues using T-Coffee (Di Tommaso *et al.*, 2011). (**B**) Phylogenetic tree of the 15 TagF-like proteins analyzed, generated using the ClustalW2-Phylogeny Service .

Di Tommaso, P., Moretti, S., Xenarios, I., Orobitg, M., Montanyola, A., Chang, J.M., Taly, J.F., and Notredame, C. 2011. T-Coffee: a web server for the multiple sequence alignment of protein and RNA sequences using structural information and homology extension. Nucleic Acids Res. 39:W13-17.

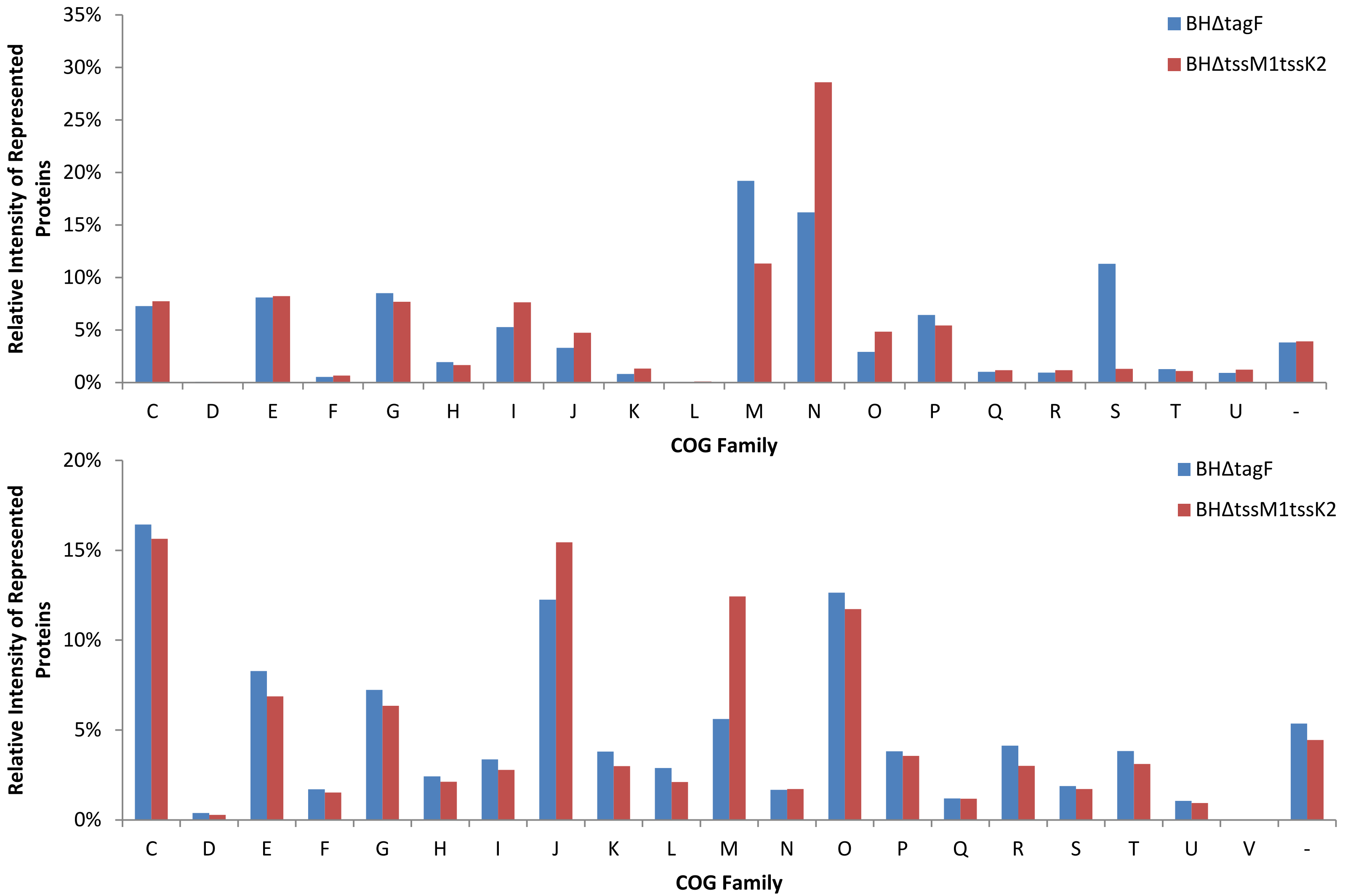

Supplementary Figure S5 │ Proteins identified by mass spectrometric analysis in *Azoarcus* mutants were grouped according to their COG family, and the relative contribution of each COG family to the overall composition of proteins was calculated for the hypersecreting mutant (BHΔtagF) and the T6SS$^-$ mutant (BHΔtssM1tssK2). **(A)** Distribution of proteins in the supernatant fraction; **(B)** Distribution of proteins in the cellular fraction.

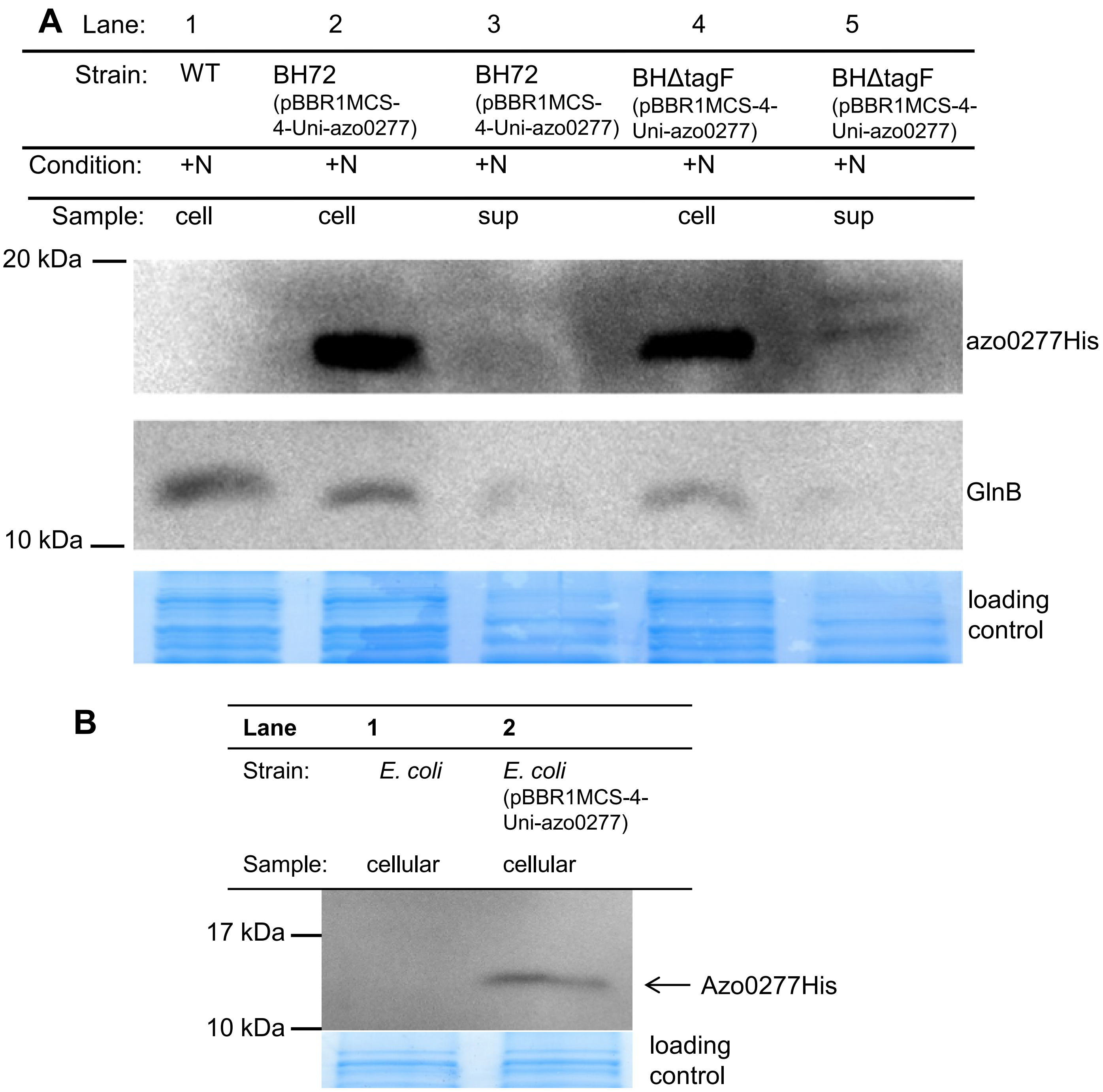

Supplementary FIGURE S6 │Western Blot analysis for abundance of Azo0277His in cellular and supernatant fractions of *Azoarcus olearius* strain BH72 (pBBR1MCS-4-Uni-azo0277) and the hypersecretion mutant BHΔtagF (pBBR1MCS-4-Uni-azo0277) (A), and in the cellular fraction of *E. coli* DH5αF´ (pBBR1MCS-4-Uni-azo0277) (B). Azo0277His was detected with a mouse anti-His antibody. Cultures were grown to an OD_{578} of 0.4 under aerobic conditions in SM medium with nitrogen source. GlnB antibodies were used to test for cellular contaminations of the supernatant fraction. Total proteins were stained with colloidal coomassie to control for equal loading. Protein mass as indicated (in kDa).

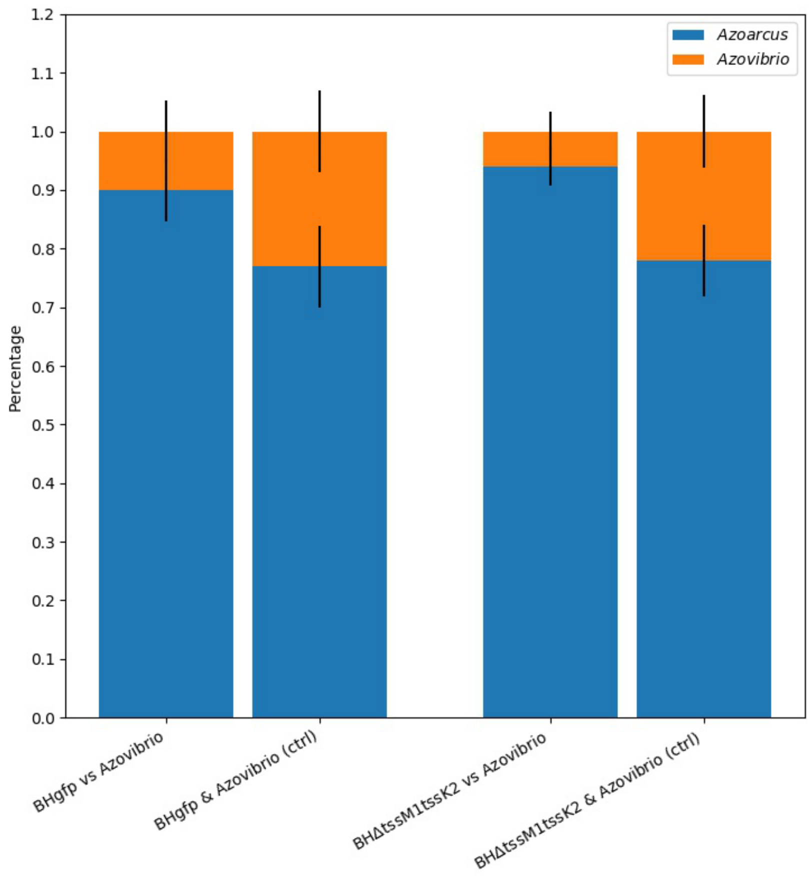

Supplementary Figure S7 | Bacterial competition assay. Relative abundance of *A. olearius* strains BHgfp (gfp-tagged wild type) and BHΔtssM1tssK2 after 24 h of co-incubation with *Azovibrio restrictus* S5B2^{T} under nitrogen-fixing, microaerobic conditions in SM agar without nitrogen source, as determined by life cell counts. At least three biological replicates with two technical repetitions were carried out for each group. In the control group for growth (Ctrl), cells were incubated separately under the same conditions and counted by life cell counts, in order to demonstrate that dominance of *Azoarcus* is related to better growth and not to killing; vs., versus. Bars, standard deviations

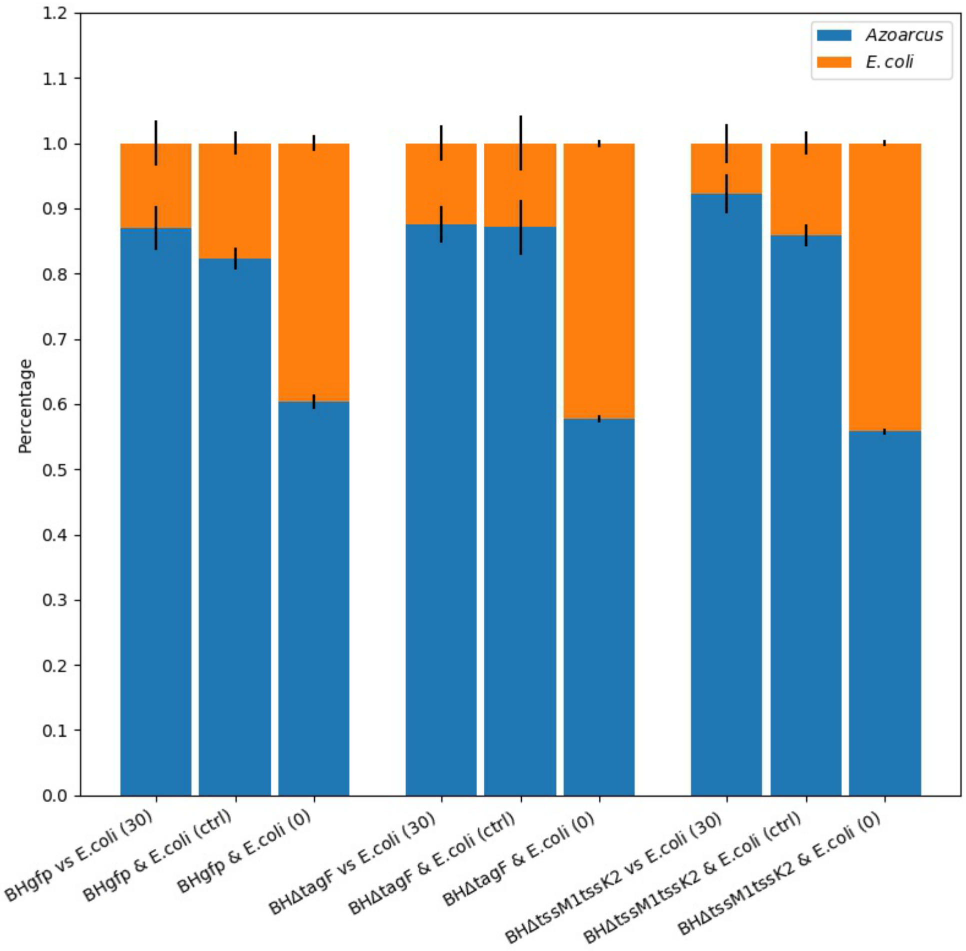

Supplementary Figure S8 │ Bacterial competition assay. Relative abundance of *Azoarcus* strains BHgfp, BHΔtagF and BHΔtssM1tssK2 after 24h co-incubation with *E.coli* DH5alpha under nitrogen-fixing, microaerobic conditions in SM agar without nitrogen source at 30°C. More than three replicates were performed for each group. In the control group (ctrl) cells were incubated separately under the same conditions to assess growth behavior. In the "0" group, the cells were mixed but kept on ice for 24 hours to control inoculation density and survival. Bars, standard deviations.

Supplementary TABLE S1 | Gene loci, previous and current protein designation

T6SS-1			T6SS-2		
**Gene Locus**	**Prev. Nomenclature**	**T6SS Nomenclature**	**Gene Locus**	**Prev. Nomenclature**	**T6SS Nomenclature**
azo1297	ImpF	TssE1	*azo3876*	VgrG-3	TssI3
azo1298	ImpK	TssL1	*azo3884*	Fha1	TagH
azo1299	ImpJ	TssK1	*azo3885*	PppA	TagG
azo1300	VasD	TssJ1	*azo3888*	PpkA	TagE
azo1301	-	-	*azo3889*	SciT, ImpM	TagF
azo1302	ImpL	TssM1	*azo3890*	ImcF	TssM2
azo1303	ImpB	TssB1	*azo3891*	OmpA2	TssL2
azo1304	ImpC	TssC1	*azo3892*	SciO	TssK2
azo1305	Hcp1	TssD1	*azo3893*	VasD	TssJ2
azo1306	-	TagL1	*azo3894*	SciA	TssA2
azo1307	VgrG1	TssI1	*azo3895*	SciH	TssB2
azo1308	ImpG	TssF1	*azo3896*	SciI	TssC2
azo1309	ImpH	TssG1	*azo3897*	SciK, Hcp2	TssD2
azo1310	ImpA	TssA1	*azo3898*	SciM, Hcp3	TssD3
azo1311	-	-	*azo3899*	SciE	TagJ
			azo3900	SciD	TssE2
			azo3901	SciC	TssF2
			azo3902	SciB	TssG2
			azo3903	ClpB2	TssH2

Supplementary TABLE S2 │ Strains and plasmids used in this study

Strain or plasmid	Relevant characteristic(s)	Reference
Strains		
Escherichia coli		
DH5αF′	F′/ φ80lacZΔM15Δ(lacZYA-argF) U169 *recA1 endA1 hsdR17* (rK–, mK+) phoA supE44 λ– thi-1 gyrA96 relA1	Invitrogen, Karslruhe, Germany
DH5αF′ (pRK2013)	DH5αF′ containing the helper plasmid pRK2013 with tra+, ColE1 replicon, Km^r	(Figurski and Helinski, 1979)
S17-1	Sp^r, MM294, RP4-2-Tc::Mu-Km::Tn7 chromosomally integrated	(Simon et al., 1983)
Azoarcus olearius	wild type strain and mutants of it	
BH72	wild type strain	(Reinhold et al., 1986)
BHΔtagF	*in-frame* deletion (bp 35-603) of *tagF via* double homologous recombination using the vector pKmsB-azo3889UD	This study
BHΔIE	Sm^r/Spc^r, marker exchange mutant of the genes *azo3470-azo3474* by double homologous recombination using the vector pK18msB-azo3470UOD	This study
BHΔtssM1tssK2	Km^r, *in-frame* deletion (bp 408-3423) of *tssM1* in mutant BHtssK2 *via* double homologous recombination using the vector pK18msBazoUpDown1302	This study
BHΔtagG	*in-frame* deletion (bp 208-686) of *tagG via* double homologous recombination using the vector pK18msBH3885	This study
BHΔfliC3	978 bp *in-frame* deletion of *fliC3*	(Buschart et al., 2012)
BHtssM1	Km^r, plasmid pK18TimpL chromosomally integrated into the genome	This study
BHtssK1	Km^r, plasmid pK18TimpJ chromosomally integrated into the genome	This study
BHtssK2	Km^r, plasmid pK18TsciO chromosomally integrated into the genome	This study
BH3472	Km^r, plasmid pKazo3472 chromosomally integrated into the genome	This study
BHtssM2Km	Km^r, marker exchange mutant of the gene *tssM2* by double homologous recombination using the vector pK18msBA-3890UKmD	This study
BHΔtss1	Sm^r/Spc^r, marker exchange mutant of the genes *azo1297* (tssE1)-*azo1311* by double homologous recombination using the vector pK18msB-Imp-UOD	This study
BHtagE	Km^r, plasmid pK18GGSTazo3888 chromosomally integrated into the genome	This study

BHΔhcp2-3	1220 bp in-frame deletion of both *hcp2(tssD2)* and *hcp3(tssD3)* in the *tss2* gene cluster	This study
BHgfp	Wild type strain BH72 carrying constitutively expressed *gfp* integrated intergenically downstream of *azo1129*	(Bachmann, 2013)
Environmental strains		
Azovibrio restricticus S5b2^T	wild type strain	(Reinhold-Hurek et al., 1993)
Azoarcus tolulyticus Td-1	wild type strain	(Zhou et al., 1995)
Plasmids		
pHP45Ω	Ap^r, Sm^r/Spc^r, derivative of vector pHP45, Sm^r/Spc^r cassette	(Prentki and Krisch, 1984)
pK18mobsacB	Km^r, RP4 *mob* region, containing *sacB* gene	(Schäfer et al., 1994)
pK18GGST	Km^r, derivative of the mobilizable cloning vector pK18mob2, promoterless *gfp* and *uidA*, T4 transcription terminator	(Krause et al., 2011)
pBBR1MCS-4	Ap^r, *lacZα*, pBBR replicon, *mob* region	(Kovach et al., 1995)
pBBR1-4gusA	Ap^r, promotorless *gusA* fragment amplified form pK18GGST with *Xho*I and *Kpn*I restriction sites (1859 bp) cloned into *Xho*I/ *Kpn*I sites pBBR1MCS-4 against *lac* promoter	(Harten, 2016)
pKmsB-azo3889UD	Km^r, fragments upstream (926 bp) and downstream (884 bp) of *azo3889*, including 34 bp at the 5' end and 99 bp at the 3' end of *azo3889*, cloned into the *Hin*dIII/*Sal*I and *Sal*I/*Xba*I sites of pK18mobsacB, respectively	This study
pK18ms-BH3885	Km^r, fragments upstream (782 bp) and downstream (983 bp) of *azo3889*, including 207 bp at the 5' end and 84 bp at the 3' end of *azo3885*, cloned into the *Eco*RI/*Sac*I and SacI/XbaI sites of pK18mobsacB, respectively	This study
pKmsB-azo3470UOD	Km^r, Sm^r/Spc^r, fragment upstream (995 bp) of *azo3470* and fragment downstream (1016 bp) of *azo3474*, cloned into *Bam*HI/*Hin*dIII and *Hin*dIII/*Nhe*I sites of pK18mobsacB, respectively, and Ω fragment of pHP45Ω cloned into the *Hin*dIII site of pK18mobsacB	This study
pK18msBazoUpDown1302	Km^r, fragments upstream (801 bp) and downstream (823 bp) of *azo1302*, including 408 bp of the 5' end and 270 bp at the 3' end of *azo1302*, cloned into *Eco*RI/*Bam*HI and *Bam*HI/*Hin*dIII sites of pK18mobsacB, respectively	This study
pK18TimpL	Km^r, fragment of *azo1302* (720 bp, starting 45 bp of the 5' end of *azo1302*) cloned into the *Xba*I/*Hin*dIII sites of pK18GGST	This study

pK18TimpJ	Km^r, fragment of *azo1299* (731 bp, starting 50 bp of the 5' end of *azo1299*) cloned into the *Xba*I/*Hin*dIII sites of pK18GGST	This study
pK18TsciO	Km^r, fragment of *azo3892* (733 bp, starting 24 bp of the 5' end of *azo3892*) cloned into the *Xba*I/*Hin*dIII sites of pK18GGST	This study
pK18GGST-azo3472	Km^r, fragment of *azo3472* (638 bp, including 303 bp of the 5' end of *azo3472*) cloned into the *Xba*I/*Hin*dIII sites of pK18GGST	This study
pK18msBA-3890UKmD	Ap^r, Km^r, fragments upstream (1012 bp) and downstream (966 bp) of *azo3890*, including 90 bp of the 5' end and 81 bp of the 3' end of *azo3890* as well as the *nptII* gene cloned into the *Eco*RI site of pK18mobsacBA	This study
pK18msB-Imp-UOD	Km^r, Sm^r/Spc^r, fragment upstream of *azo1297* (1070 bp, including 19 bp of the 5' end of *azo1297*) and fragment downstream of *azo1311* (1083 bp, including 161 bp of the 3' end of *azo1311*), cloned into the *Xba*I/*Bam*HI and *Bam*HI/*Sma*I sites and Ω fragment of pHP45Ω cloned into the *Bam*HI site of pK18mobsacB	This study
pK18GGSTazo 3888	Km^r, fragment of azo3888 (bp 91-644) cloned into the *Xba*I-*Hin*dIII sites of pK18GGST	This study
pk18msB- Up Downhcp2&3	Km^r, fragments upstream of *azo3897* (1362 bp including 306 bp of the 3' end of *azo3896*, and downstream of *azo3898* (1249 bp including 307 bp the 3' end of *azo3899*), cloned into *Bam*HI/*Xba*I and *Xba*I/*Hin*dIII sites of pK18mobsacB	This study
pBBRI-gusA_TagF	Ap^r, 7828 bp, promoter fragment of *tssJ2* (375 bp with terminal *Xba*I/*Hin*dIII restriction sites) and *tagF* (704 bp with terminal *Hin*dIII/*Xho*I restriction sites) cloned into *Xba*I/*Xho*I sites of pBBR1-4gusA	This study
pBBR1MCS-4-Uni-azo0277	Ap^r, 5519 bp, *azo0277* (417 bp with terminal restriction sites *Bam*HI/*Xba*I) preceded by a constitutive promoter Unibrick (59 bp with terminal *Hin*dIII/*Bam*HI restriction sites), cloned into pBBR1MCS-4 (*Hin*dIII/*Xba*I) providing hexahistidine codons CAC at then 3' end of the gene	Klein (2014)
pJet1.2-azo1129up+down	Amp^r, fragments upstream (962 bp) and downstream (993 bp) of the 3' end of azo1129, cloned into the *Sal*I/*EcoR*I and *EcoR*I/*Xba*I sites of pJet1.2	Bachmann 2013
pJet1.2-azo1129-Pbrick-gfp	Amp^r, fragments of *gfp* and promoter inserted into the *EcoR*I site of pJet1.2-azo1129up+down	Bachmann 2013
pK18mobsacB-azo1129-Pbrick-gfp	Km^r, the insert of pJet1.2-azo1129-Pbrick-gfp from pJet1.2-azo1129-Pbrick-gfp inserted into the *Sal*I/*Xba*I site of pK18mobsacB	Bachmann 2013

Bachmann A (2013) Integration eines stabilen Fluoreszenzmarkers in *Azoarcus* sp. Stamm BH72. Bachelor thesis, University of Bremen.

Buschart, A., Sachs, S., Chen, X., Herglotz, J., Krause, A., and Reinhold-Hurek, B. (2012). Flagella mediate endophytic competence rather than act as MAMPS in rice - *Azoarcus* sp. strain BH72 interactions. *Mol. Plant-Microbe Interact.* 25**,** 191–199.

Figurski, D.H., and Helinski, D.R. (1979). Replication of an origin-containing derivative of plasmid RK2 dependent on a plasmid function provided in *trans*. *Proc Natl Acad Sci U S A* 76**,** 1648-1652.

Harten, T. (2016). Analysis of the promoter region of *exaA3* encoding an alcohol dehydrogenase in *Azoarcus* sp. BH72. Master thesis, University of Bremen, Faculty of Biology and Chemistry.

Klein, K. Analysis of protein secretion by the Type VI secretion system in *Azoarcus* sp. BH72. Master thesis, University of Bremen, Faculty of Biology and Chemistry.

Kovach, M.E., Elzer, P.H., Hill, D.S., Robertson, G.T., Farris, M.A., Roop, R.M., II, et al. (1995). Four new derivatives of the broad-host-range cloning vector pBBR1MCS, carrying different antibiotic-resistance cassettes. *Gene* 166**,** 175-176.

Krause, A., Leyser, B., Miché, L., Battistoni, F., and Reinhold-Hurek, B. (2011). Exploring the function of alcohol dehydrogeanses during the endophytic life of *Azoarcus* sp. strain BH72. *Mol. Plant-Microbe Interact.* 24**,** 1325-1332.

Prentki, P., and Krisch, H.M. (1984). *In vitro* insertional mutagenesis with a selectable DNA Fragment. *Gene* 29**,** 303-314.

Reinhold-Hurek, B., Hurek, T., Gillis, M., Hoste, B., Vancanneyt, M., Kersters, K., et al. (1993). *Azoarcus* gen. nov., nitrogen-fixing proteobacteria associated with roots of Kallar grass (*Leptochloa fusca* (L.) Kunth) and description of two species *Azoarcus indigens* sp. nov. and *Azoarcus communis* sp. nov. *Int. J. Syst. Bacteriol.* 43**,** 574-584.

Reinhold, B., Hurek, T., Niemann, E.-G., and Fendrik, I. (1986). Close association of *Azospirillum* and diazotrophic rods with different root zones of Kallar grass. *Appl. Environ. Microbiol.* 52**,** 520-526.

Schäfer, A., Tauch, A., Jager, W., Kalinowski, J., Thierbach, G., and Puhler, A. (1994). Small mobilizable multi-purpose cloning vectors derived from the *Escherichia coli* plasmids pK18 and pK19: selection of defined deletions in the chromosome of *Corynebacterium glutamicum*. *Gene* 145**,** 69-73.

Simon, R., Priefer, U., and Pühler, A. (1983). A broad host range mobilisation system for *in vivo* genetic engineering: Transposon mutagenesis in gram negative bacteria. *Nat. Biotechnol.* 1 (9)**,** 784-791.

Zhou, J., Fries, M.R., Chee-Sanford, J.C., and Tiedje, J.M. (1995). Phylogenetic analyses of a new group of denitrifiers capable of anaerobic growth on toluene and description of *Azoarcus tolulyticus* sp. nov. *Int. J. Syst. Bacteriol.* 45**,** 500-506.

Supplementary TABLE S3 │Primers designed and used in this study

Primer	Sequence (5' - 3')[1,2]
up3889for	ag-*aagctt*(*Hin*dIII)-GCGGTGGTGACGCTGCTCAACGAT
up3889rev	ga-*gtcgac*(*Sal*I)-GTACCAGCCGGGGGCGCTTTCG
down3889for	gc-*gtcgac*(*Sal*I)-GCGGCGGATGGATTCGCGTGCCA
down3889rev	ca-*tctaga*(*Xba*I)-CCGCCAGCGCATACACGTCGGT
up3470for	tgc-*ggatcc*(*Bam*HI)-GCGCAGAAGATCCCGACCAT
up3470rev	ccg-*aagctt*(*Hin*dIII)-CGTATCGGGAAAGCCGAGAG
down3470for	ccg-*aagctt*(*Hin*dIII)-GAACGGCGCCTTGTGACAG
down3470rev	ctg-*gctagc*(*Nhe*I)-CAGAACATGCGCACCAACCA
up1302for	gct-*gaattc*(*Eco*RI)-GGGTGAAGGAGAGGTGGAC
up1302rev	gtc-*ggatcc*(*Bam*HI)-CTGCTTGATCAAAGCGGTCT
downforC	gtt-*ggatcc*(*Bam*HI)-CAGACCTGGGCGAGTTTCAGT
downrev4real	gcg-*aagctt*(*Hin*dIII)-CTTCGTTGAGCAGGGCTTC
impL-F	gc-*tctaga*(*Xba*I)-taaATGGTGCTTGTCCAGACCTT
impL-R	ccc-*aagctt*(*Hin*dIII)-ATCACGTTCGGATGGCAAAGC
impJ-F	gc-*tctaga*(*Xba*I)-taaatGCAGCACTTCCAGCAGCAG
impJ-R	cc-*aagctt*(*Hin*dIII)-GCTGCAGACGGGCGTAGTT
sciO-F	gc-*tctaga*(*Xba*I)-taaATGTCCGAAGGGCTGTTCCT
sciO-R	ccc-*aagctt*(*Hin*dIII)-GTTCGAGGTGGGCCATCAC
azo3472for	gcc-*tctaga*(*Xba*I)-CGGCTGACCGACAAGGATTG
azo3472rev	tcc-*aagctt*(*Hin*dIII)-CGGGTGTTTCCTGACTTGGG
3890UpFor	gtg-*tctaga*(*Xba*I)-GTTCCACAACGAAGCCTGGG
3890UpRev	cga-*gtcgac*(*Sal*I)-ACCGACGAACCAGATCACCA
3890DownFor	agc-*gtcgac*(*Sal*I)-TTCGAAGTGACCACCAACAGC
3890DownRev	gca-*catatg*(*Nde*I)-ATTCCTTGAGTGCGACCTTGC
ImpUpFor	cg-*tctaga*(*Xba*I)-GATGACACCGCCAAGGTGAA
ImpUpRev	ga-*ggatcc*(*Bam*HI)-CTACCGCGTAAAGCCCTGAA
ImpDownFor	tg-*cctagg*(*Bam*HI)-TCGCCTTCGATGATGACAGC
ImpDownRev	ag-*cccggg*(*Sma*I)-GACGACTTGCTCATCTCGGT
3885upF	aaa-*gaattc*(*Eco*RI)-CCGAACTGATGGAGCACAT
3885upR	aaa-*tctgag*(*Sac*I)-GGCTTCGCTCACTTCGGGATG
3885dnF	aaa-*gagctc*(*Sac*I)-GACAACCACCACAGCGATAA
3885dnR	aaa-*tctgag*(*Xba*I)-AGCACCAGGCTGAAGTTCT
3888(551)F	ggg-*tctaga*(*Xba*I)-TAAGCTGGGCGAATTCGAGAT
3888(551)R	ggg-*aagctt*(*Hin*dIII)-GGCTTGAGGATGACCGTAAG
azo3897F	cg-*ggatcc*(*Bam*HI)-cgTCCACCACGAGGACTTCCAGAAAC
azo3897R	gc-*tctaga*(*Xba*I)-gcTCCTGAATGATGTCGGATGCTGC
azo3898F	gc-*tctaga*(*Xba*I)-gcCGCCCCAATAAGCCGAGGAG
azo3898R	cc-*aagctt*(*Hin*dIII)-ggCCGAGATGCGCACGCTGATC
XbaI_Promoter_F	gc-*tctaga*(*Xba*I)-gcGCCTCTTCCATCTGGAGGAAGTC
HindIII_promoter_R	cc-*aagctt*(*Hin*dIII)-ggAATCTCCTTGCCCTATTACATTCG
HindIII_TagF_F	cc-*aagctt*(*Hin*dIII)-ggGCATGCTGCCCGGCGAAAG
XhoI_TagF_R	ccg-*ctcgag*(*Xho*I)-cggCTAAGCCTGGCCCGGCGC

[1] Restriction sites present in the primers are in italics with enzyme name in parenthesis.
[2] Homologous regions of the primers highlighted with capital letters.

Supplementary TABLE S4 │ Proteome of *A. olearius* - Distribution of different COG categories based on the relative protein intensity of proteins grouped in the respective COG categories

	Cellular protein composition[1]		Supernatant protein composition	
COG category	BHΔtagF	BHΔtssM1tssK2	BHΔtagF	BHΔtssM1tssK2
C – energy production and conversion	16,43%[2]	15,64%	7,27%	7,74%
D – cell cycle control, cell division and chromosome partitioning	0,39%	0,28%	0,08%	0,07%
E – amino acid transport and metabolism	8,29%	6,87%	8,10%	8,24%
F – nucleotide transport and metabolism	1,70%	1,53%	0,54%	0,67%
G – carbohydrate transport and metabolism	7,23%	6,35%	8,52%	7,71%
H – coenzyme transport and metabolism	2,43%	2,13%	1,94%	1,66%
I – lipid transport and metabolism	3,37%	2,78%	5,28%	7,63%
J – translation, ribosomal structure and biogenesis	12,26%	15,45%	3,30%	4,74%
K – transcription	3,80%	2,99%	0,83%	1,34%
L – replication, recombination and repair	2,88%	2,12%	0,05%	0,10%
M – cell wall/membrane/envelope biogenesis	5,62%	12,43%	19,21%	11,34%
N – cell motility	1,68%	1,73%	16,20%	28,59%
O – post-translational modification, protein turnover, and chaperones	12,65%	11,73%	2,94%	4,85%
P – inorganic ion transport and metabolism	3,82%	3,57%	6,45%	5,44%
Q – secondary metabolites biosynthesis, transport, and catabolism	1,20%	1,18%	1,02%	1,18%
R – general function prediction only	4,13%	3,01%	0,96%	1,18%
S – function unknown	1,88%	1,71%	11,32%	1,30%
T – signal transduction mechanisms	3,83%	3,11%	1,27%	1,10%
U – intracellular trafficking, secretion, and vesicular transport	1,06%	0,95%	0,92%	1,22%
V – defense mechanisms	0,01%	0,01%	0,00%	0,00%
no COG category annotated	5,36%	4,44%	3,83%	3,91%

[1] Distribution was analyzed individually for both mutants, *Azoarcus olearius* sp. BHΔtagF and BHΔtssM1tssK2, and with regards to the two different sample types (cellular proteins and supernatant proteins).
[2] Data based on results obtained by LC-MS/MS analysis of total cellular proteins and supernatant proteins in 4 independent replicates for each mutant.

Supplementary TABLE S5 │ Proteins identified as differentially abundant, Part 1

Azo Number	UniProt	Description	FC cells	p-value	FC sup	p-value	Predicted Localization
**Motility and chemotaxis related proteins**							
azo0091	A1K1K4	Probable methyl-accepting chemotaxis protein	-9.90	0.005	-	-	Cytoplasmic Membrane
azo0406	A1K2G8	Putative methyl-accepting chemotaxis protein	-8.60	0.003	-	-	Cytoplasmic Membrane
azo0407	A1K2G9	Putative methyl-accepting chemotaxis protein	-8.78	0.003	-	-	Cytoplasmic Membrane
azo2547	A1K8K9	Probable methyl-accepting chemotaxis protein	-8.46	0.003	-	-	Cytoplasmic Membrane
azo2705	A1K916	Probable flagellar protein	-	-	-4.91	0.011	Unknown
azo2706	A1K917	Flagellar hook-associated protein	-	-	-6.15	0.023	Extracellular
azo2721	A1K932	Putative flagellar hook-length control protein FliK	-	-	-3.03	0.017	Extracellular
azo2730	A1K941	Flagellar hook-filament junction protein 3	-	-	-3.33	0.040	Extracellular
azo2731	A1K942	Flagellar hook-filament junction protein 1	-	-	-2.89	0.043	Extracellular
azo2735	A1K946	Flagellar basal-body rod protein FlgG	-	-	-2.07	0.009	Extracellular
azo2736	A1K947	Flagellar basal-body rod protein FlgF	-	-	-2.52	0.034	Periplasmic
azo2738	A1K949	Probable basal-body rod modification protein FlgD	-	-	-2.13	0.014	Extracellular
azo2917	A1K9M8	Putative Tfp pilus assembly protein	-	-	-2.92	0.010	Outer Membrane
azo3456	A1KB66	Probable pilus biogenesis protein	-2.66	0.003	-	-	Cytoplasmic
azo3691	A1KBV1	Putative methyl-accepting chemotaxis transducer	-10.54	0.013	-	-	Cytoplasmic Membrane
**Interesting or T6SS related proteins**							
azo0277	A1K239	Hyothetical protein	-	-	only in BHΔ3889	-	Unknown
azo0777	A1K3I9	Hypothetical secreted protein	-	-	-3.40	0.014	Cytoplasmic Membrane
azo1089	A1K4F1	Conserved hypothetical lipoprotein	-	-	4.95	0.027	Outer Membrane
azo2271	A1K7T3	Uncharacterized protein	-	-	-2.59	0.009	Unknown
azo2584	A1K8P6	Uncharacterized protein	-	-	-3.59	0.021	Outer Membrane
azo3190	A1KAF1	Uncharacterized protein	-6.95	0.001	-	-	Unknown
azo3876	A1KCD6	Uncharacterized protein; TssI3, VgrG3	-	-	96.13	0.002	Cytoplasmic
azo3897	A1KCF7	Putative cytoplasmic protein,sciM; Hcp2, TssD2	-	-	48.73	0.001	Extracellular
azo3898	A1KCF8	Putative cytoplasmic protein,sciK; Hcp3, TssD3	-	-	23.85	0.000	Extracellular

Azo Number	UniProt	Description	FC cells	p-value	FC sup	p-value	Predicted Localization
**Ribosomal proteins**							
azo1136	A1K4J8	50S ribosomal protein L28	-4.75	0.007	-	-	Cytoplasmic
azo2104	A1K7B6	30S ribosomal protein S15	-3.33	0.014	-	-	Cytoplasmic
azo2760	A1K971	50S ribosomal protein L13	-4.13	0.000	-	-	Cytoplasmic
azo2898	A1K9K9	30S ribosomal protein S16	-3.42	0.003	-	-	Cytoplasmic
azo2901	A1K9L2	50S ribosomal protein L19	-3.64	0.001	-	-	Cytoplasmic
azo3168	A1KAC9	50S ribosomal protein L27	-2.70	0.018	-	-	Cytoplasmic
azo3392	A1KB02	30S ribosomal protein S4	-3.27	0.001	-	-	Cytoplasmic
azo3394	A1KB04	30S ribosomal protein S13	-5.57	0.006	-	-	Cytoplasmic
azo3398	A1KB08	50S ribosomal protein L15	-3.87	0.001	-	-	Cytoplasmic
azo3400	A1KB10	30S ribosomal protein S5	-2.99	0.001	-	-	Cytoplasmic
azo3401	A1KB11	50S ribosomal protein L18	-3.47	0.003	-	-	Cytoplasmic
azo3402	A1KB12	50S ribosomal protein L6	-4.44	0.007	-	-	Cytoplasmic
azo3405	A1KB15	50S ribosomal protein L5	-2.47	0.006	-2.88	0.005	Cytoplasmic
azo3407	A1KB17	50S ribosomal protein L14	-4.05	0.011	-	-	Cytoplasmic
azo3410	A1KB20	50S ribosomal protein L16	-3.37	0.008	-	-	Cytoplasmic
azo3411	A1KB21	30S ribosomal protein S3	-4.84	0.000	-	-	Cytoplasmic
azo3412	A1KB22	50S ribosomal protein L22	-3.18	0.002	-	-	Cytoplasmic
azo3414	A1KB24	50S ribosomal protein L2	-4.67	0.000	-	-	Cytoplasmic
azo3417	A1KB27	50S ribosomal protein L3	-3.85	0.000	-	-	Cytoplasmic
azo3421	A1KB31	30S ribosomal protein S7	-8.94	0.005	-	-	Cytoplasmic
azo3428	A1KB38	50S ribosomal protein L11	-2.55	0.014	-	-	Cytoplasmic
**Other proteins**							
azo0095	A1K1K8	DNA topoisomerase (EC 5.99.1.2)	-2.18	0.003	-	-	Cytoplasmic
azo0101	A1K1L4	Protease HtpX homolog (EC 3.4.24.-)	-5.14	0.000	-	-	Cytoplasmic Membrane
azo0155	A1K1R8	ATP synthase subunit b (ATP synthase F(0) sector subunit b) (ATPase subunit I) (F-type ATPase subunit b) (F-ATPase subunit b)	-4.15	0.001	-	-	Cytoplasmic Membrane
azo0187	A1K1V0	Conserved hypothetical cytochrome c5	-3.07	0.009	-	-	Unknown
azo0204	A1K1W6	Probable lipoprotein	-	-	-2.43	0.024	Periplasmic
azo0247	A1K209	Bacterioferritin	-3.45	0.019	-	-	Cytoplasmic

Azo Number	UniProt	Description	FC cells	p-value	FC sup	p-value	Predicted Localization
azo0415	A1K2H7	Conserved hypothetical secreted protein	-	-	4.92	0.042	Unknown
azo0416	A1K2H8	Putative peptidoglycan-associated lipoprotein	-10.56	0.017	-	-	Outer Membrane
azo0480	A1K2P2	Conserved hypothetical iron-sulfur binding oxidase	-3.56	0.000	-	-	Unknown
azo0507	A1K2R9	Protein RecA (Recombinase A)	-	-	-12.19	0.003	Cytoplasmic
azo0587	A1K2Z9	Adenosylhomocysteinase (EC 3.3.1.1) (S-adenosyl-L-homocysteine hydrolase)	-	-	-2.14	0.025	Cytoplasmic
azo0607	A1K319	Putative TonB-dependent receptor	-2.01	0.001	-	-	Outer Membrane
azo0685	A1K397	Probable Methylmalonyl-CoA mutase large subunit (EC 5.4.99.2)	-	-	-2.54	0.016	Cytoplasmic
azo0904	A1K3W6	Protein translocase subunit SecD	only in BHΔimpLsciO	-	-	-	Cytoplasmic Membrane
azo0962	A1K424	Conserved hypothetical ubiquinol-cytochrome c reductase cytochrome c1 protein	-2.32	0.001	-	-	Cytoplasmic Membrane
azo1012	A1K474	Putative glycerol-3-phosphate-binding periplasmic protein	-	-	2.12	0.006	Periplasmic
azo1063	A1K4C5	Chaperone protein DnaK (HSP70) (Heat shock 70 kDa protein) (Heat shock protein 70)	-	-	-2.13	0.001	Cytoplasmic
azo1344	A1K556	Cbb3-type cytochrome c oxidase subunit	-7.30	0.001	-	-	Unknown
azo1549	A1K5R1	Succinate dehydrogenase (EC 1.3.99.1)	-4.31	0.015	-	-	Cytoplasmic Membrane
azo1555	A1K5R7	Probable 2-oxoglutarate dehydrogenase (EC 1.2.4.2)	-2.03	0.004	-	-	Cytoplasmic
azo1566	A1K5S8	Probable ATP-dependent Clp protease, ATP-binding subunit ClpB	-	-	-2.59	0.008	Cytoplasmic
azo2072	A1K784	Trigger factor (TF) (EC 5.2.1.8) (PPIase)	-	-	-2.51	0.018	Cytoplasmic
azo2108	A1K7C0	Putative N utilization substance protein A	-	-	-2.25	0.010	Cytoplasmic
azo2153	A1K7G5	Conserved hypothetical secreted protein	-	-	2.30	0.022	Unknown
azo2155	A1K7G7	Conserved hypothetical secreted protein	-	-	3.49	0.045	Unknown
azo2156	A1K7G8	Probable TonB-dependent receptor	-	-	11.22	0.043	Outer Membrane
azo2171	A1K7I3	Putative ribose and galactose chemoreceptor protein	-9.46	0.006	-	-	Cytoplasmic Membrane
azo2269	A1K7T1	Conserved hypothetical band 7 family protein	-3.75	0.004	-	-	Cytoplasmic
azo2340	A1K802	Dienelactone hydrolase family protein	-	-	3.26	0.002	Cytoplasmic
azo2396	A1K858	Putative TonB-dependent receptor	-	-	2.30	0.009	Unknown
azo2588	A1K8Q0	Outer membrane protein A	-6.27	0.019	-	-	Outer Membrane
azo2795	A1K9A6	Putative periplasmic substrate binding protein	-3.70	0.000	-	-	Unknown

Azo Number	UniProt	Description	FC cells	p-value	FC sup	p-value	Predicted Localization
azo3154	A1KAB5	Acetolactate synthase (EC 2.2.1.6)	-	-	-2.27	0.026	Cytoplasmic
azo3239	A1KAJ9	Aspartate--tRNA ligase (EC 6.1.1.12) (Aspartyl-tRNA synthetase) (AspRS)	-	-	-2.12	0.032	Cytoplasmic
azo3283	A1KAP3	Conserved hypothetical polysaccharide export protein	-	-	2.24	0.000	Cytoplasmic Membrane
azo3290	A1KAQ0	Outer membrane porin protein	-3.37	0.006	2.16	0.004	Outer Membrane
azo3291	A1KAQ1	Outer membrane porin protein	-2.91	0.010	2.10	0.016	Outer Membrane
azo3555	A1KBG5	TonB-dependent receptor, putative	-	-	2.18	0.017	Outer Membrane
azo3556	A1KBG6	Putative TonB-dependent receptor	-	-	2.58	0.004	Outer Membrane
azo3638	A1KBP8	Conserved hypothetical phosphate acetyltransferase (EC 2.3.1.8)	-	-	-2.01	0.031	Cytoplasmic
azo3738	A1KBZ8	Conserved hypothetical secreted protein	-	-	2.02	0.041	Outer Membrane
azo3960	A1KCM0	Putative alpha helix protein,yicC	-	-	-2.50	0.011	Cytoplasmic

FC: Fold change, given as BHΔtagF/BHΔtssM1tssK2

p-value according to 2-sided Student's t-test

Localization according to PsortB database

BLAST results considered hits with E value e-5 or lower

Pfam Domains according to Pfam.Sanger.Ac.UK

Transmembrane Helix (TMH) as predicted by CBS TMH Prediction Service

Signal Peptide as predicted by CBS Signal Peptide Prediction Service

Supplementary TABLE S5 | Proteins identified as differentially abundant, Part 2 Domains

Azo Number	Related Proteins	E value	Organism	Pfam Domains	TMH	Signal Peptide
**Motility and chemotaxis related proteins**						
azo0091	methyl-accepting chemotaxis protein	0	Thauera sp. 63	HAMP; MCPsignal	2	No
azo0406	methyl-accepting chemotaxis sensory transducer	0.00E+00	Thauera sp. 28	Cache_2; HAMP; MCPsignal	2	No
azo0407	methyl-accepting chemotaxis sensory transducer	0.00E+00	Geobacter levleyi SZ	4HB_MCP_1; HAMP; MCPsignal	1	No
azo2547	methyl-accepting chemotaxis protein	0.00E+00	Thauera sp. 63	4HB_MCP_1; HAMP; MCPsignal	2	No
azo2705	flagellar protein	3.00E-40	Thauera sp. 27	FlaG	0	No
azo2706	flagellar hook-associated protein	0.00E+00	Thauera sp. 27	Flid_N; FliD_C	0	No
azo2721	flagellar hook-length control protein	4.00E-51	Thauera sp. 63	Flg_hook	0	No
azo2730	flagellar hook-filament junction protein 3	4.00E-144	Thauera sp. 63	Flagellin_N; Flagellin_C	0	No
azo2731	flagellar hook-filament junction protein 1	0.00E+00	Thauera sp. 63	Flg_bbr_C	0	No
azo2735	flagellar basal body rod protein FlgG	2.00E-163	Thauera phenylacetica	Flg_bb_rod; Flg_bbr_C	0	No
azo2736	flagellar basal body rod protein FlgF	5.00E-133	Thauera phenylacetica	Flg_bbr_C	0	No
azo2738	flagellar basal body rod modification protein FlgD	3.00E-61	Sulfuritalea hydrogenivorans	FlgD; FLgD_tudor; FlgD_iq	0	No
azo2917	tfp pilus assembly protein	0.00E+00	Thiocystis violascens	Neisseria_PilC	0	Yes
azo3456	chemotaxis protein	0.00E+00	Aromatoleum aromaticum	PilJ; MCP_signal	0	No
azo3691	methyl-accepting chemotaxis protein	0.00E+00	Dechlorosoma suillum PS	HAMP; MCPsignal	2	No
**Interesting or T6SS related proteins**						
azo0277	hypothetical protein	-	-	-	0	No
azo0777	hypothetical protein	3.00E-26	Thauera linaloolentis	-	1	No
azo1089	peptidase M23	3.00E-91	Thauera sp. 27	LysM; Peptidase_M23	1	Yes
azo2271	histidine kinase	9.00E-123	Thauera terpenica	HDOD	0	No
azo2584	hypothetical protein	2.00E-128	Methyloversatilis discipulorum	-	0	No

Azo Number	Related Proteins	E value	Organism	Pfam Domains	TMH	Signal Peptide
azo3190	hypothetical protein	0.00E+00	Thauera linaloolentis	TPR_2; TPR_8; TPR_2; TPR_9; TPR_8; TPR_11; TPR_16; TPR_19; TPR_9; TPR_8; TPR_19	0	No
azo3876	type VI secretion protein, TssI3	0.00E+00	Polaromonas glacialis	Phage_GPD; Gp5_C; Gp5_C	0	No
azo3897	type VI secretion system effector, Hcp2, TssD2	2.00E-83	Derxia gummosa	T6SS_HCP	0	No
azo3898	type VI secretion system effector, Hcp3, TssD3	2.00E-41	Leptothrix cholodnii	T6SS_HCP	0	No
**Ribosomal proteins**						
azo1136	50S ribosomal protein L28	1.00E-46	Thauera terpenica	Ribosomal_L28	0	No
azo2104	30s ribosomal protein S15	1.00E-52	Aromatoleum aromaticum	Ribosomal_S15	0	No
azo2760	50S ribosomal protein L13	2.00E-94	Aromatoleum aromaticum	Ribosomal_L13	0	No
azo2898	30S ribosomal protein S16	4.00E-49	Thauera sp. 63	Ribosomal_S16	0	No
azo2901	50S ribosomal protein L19	6.00E-75	Aromatoleum aromaticum	Ribosomal_L19	0	No
azo3168	50S ribosomal protein L27	2.00E-48	Aromatoleum aromaticum	Ribosomal_L27	0	No
azo3392	30S ribosomal protein S4	9.00E-139	Thauera sp. 63	Ribosomal_S4; S4	0	No
azo3394	30S ribosomal protein S13	1.00E-77	Thauera linaloolentis	Ribosomal_S13	0	No
azo3398	50S ribosomal protein L15	1.00E-78	Thauera terpenica	Ribosomal_L18e	0	No
azo3400	30S ribosomal protein S5	7.00E-113	Thauera linaloolentis	Ribosomal_S5; Ribosomal_S5_C	0	No
azo3401	50S ribosomal protein L18	2.00E-69	Thauera terpenica	Ribosomal_L18p	0	No
azo3402	50S ribosomal protein L6	3.00E-102	Thauera sp. 63	Ribosomal_L6; Ribosomal_L6	0	No
azo3405	50S ribosomal protein L5	1.00E-116	Uliginosibacterium gangwonense	Ribosomal_L5; Ribosomal_L5_C	0	No
azo3407	50S ribosomal protein L14	1.00E-78	Thauera linaloolentis	Ribosomal_L14	0	No
azo3410	50S ribosomal protein L16	2.00E-88	Aromatoleum aromaticum	Ribosomal L16	0	No
azo3411	30S ribosomal protein S3	3.00E-161	Thauera phenylacetica	KH_2; Ribosomal S3_C	0	No

Azo Number	Related Proteins	E value	Organism	Pfam Domains	TMH	Signal Peptide
azo3412	50S ribosomal protein L22	4.00E-68	Thauera sp. 63	Ribosomal_L22	0	No
azo3414	50S ribosomal protein L2	7.00E-180	Thauera sp. 63	Ribosomal _L2; Ribosomal_L2_C	0	No
azo3417	50S ribosomal protein L3	3.00E-125	Thauera linaloolentis	Ribosomal_L3	0	No
azo3421	30S ribosomal protein S7	1.00E-102	Thauera sp. 63	Ribosomal_S7	0	No
azo3428	50S ribosomal protein L11	6.00E-92	Thauera linaloolentis	Ribosomal_L11_N; Ribosomal_L11	0	No
**Other proteins**						
azo0095	DNA topoisomerase III	0	Thauera sp. 28	Toprim; Topoisom_bac; Toprim_Crpt; Toprim_Crpt	0	No
azo0101	M48 family peptidase	1.00E-179	Thauera sp. 28	Peptidase_M48	4	No
azo0155	F0F1 ATP synthase subunit B	3.00E-77	Uliginosibacterium gangwonense	ATP-synt_B	1	No
azo0187	cytochrome C class I	2.00E-69	Thauera linaloolentis	Cytochrome_CBB3	0	Yes
azo0204	ABC transporter permease	6.00E-153	Cupriavidus sp. HPC(L)	Bmp	1	Yes
azo0247	bacterioferritin	2.00E-70	Rubrivivax gelatinosus	Ferritin	0	No
azo0415	tol-pal system protein YbgF	8.00E-103	Thauera terpenica	TolA_bind_tri; YfiO	0	Yes
azo0416	peptidoglycan-binding protein	3.00E-98	Thauera terpenica	OmpA	0	Yes
azo0480	FAD linked oxidase	0.00E+00	Thauera sp. 28	DUF3683; FAD_binding_4; FAD-oxidase_C; FAD-oxidase_C; Fer4_8; CCG; CCG; DUF3400	0	No
azo0507	recombinase RecA	0.00E+00	Thauera linaloolentis	RecA	0	No
azo0587	S-adenosyl-L-homocystein hydrolase	0.00E+00	Thauera linaloolentis	AdoHcyase	0	No
azo0607	TonB-dependent siderophore receptor	0.00E+00	Thauera aminoaromatica S2	Plug; TonB_dep_Rec	0	Yes
azo0685	methylmalonyl-CoA mutase	0.00E+00	Thauera linaloolentis	MM_CoA_mutase; B12-binding	0	No
azo0904	preprotein translocase subunit SecD	0	Thauera sp. 63	SecD-TM1; Sec_GG; SecD_SecF	4	No
azo0962	cytochrome C	2.00E-149	Thauera terpenica	Cytochrom_C1	1	Yes
azo1012	glycerol-3-phosphate ABC transporter substrate-binding protein	0.00E+00	Thauera terpenica	SBP_bac_8	0	Yes
azo1063	molecular chaperone DnaK	0.00E+00	Thauera sp. 63	HSP70	0	No
azo1344	cytochrome CBB3	2.00E-175	Thauera terpenica	FixP_N; Cytochrome_CBB3; Cytochrome_CBB3	2	No

Azo Number	Related Proteins	E value	Organism	Pfam Domains	TMH	Signal Peptide
azo1549	succinate dehydrogenase	3.00E-59	Thauera sp. 63	Sdh_cyt	3	No
azo1555	2-oxoglutarate dehydrogenase E1 component	0.00E+00	Thauera sp. 63	2-oxogl_dehyd_N; E1_dh; Transket_pyr; OxoGdeHyase_C	0	No
azo1566	protein disaggregation chaperone	0.00E+00	Aromatoleum aromaticum	Clp_N; Clp_N; AAA; AAA_2; ClpB_D2-small	0	No
azo2072	trigger factor	0.00E+00	Aromatoleum aromaticum	Trigger_N; FKBP_C; Trigger_C	0	No
azo2108	transcription termination factor NusA	0.00E+00	Thauera phenylacetica	NusA_N; S1; KH_5; HHH_5	0	No
azo2153	membrane protein	7.00E-60	Limnohabitans sp. Rim28	DUF461	1	Yes
azo2155	glycosyl hydrolase	4.00E-104	Ralstonia sp. UNC404CL21Col	-	0	Yes
azo2156	TonB-depdendent receptor	0.00E+00	Burkholderia cepacia	Plug; TonB_dep_Rec	1	Yes
azo2171	ribose and galactose chemoreceptor protein	0.00E+00	Azospira oryzae	4HB_MCP_1; HAMP; MCPsignal	0	No
azo2269	membrane protein	2.00E-173	Dechloromonas aromatica	Band_7	1	No
azo2340	carboxymethylenebutenolidase	2.00E-161	Methylophilaceae bacterium 11	DLH	0	No
azo2396	TonB-dependent receptor	0.00E+00	Dechloromonas aromatica	Plug; TonB_dep_Rec	0	No
azo2588	membrane protein	5.00E-132	Aromatoleum aromaticum	OmpA	0	Yes
azo2795	ABC transporter substrate-binding protein	0.00E+00	Azospira oryzae	DctP	0	No
azo3154	acetolactate synthase 3 catalytic subunit	0.00E+00	Thauera terpenica	TPP_enzyme_N; TPP_enzyme_M; TPP_enzyme_C	0	No
azo3239	aspartyl-TRNA synthetase	0.00E+00	Thauera sp. 63	tRNA_anti-codon; tRNA-synt_2; GAD	0	No
azo3283	sugar ABC transporter substrate-binding protein	3.00E-117	Thauera linaloolentis	Poly_export; SLBB	1	No
azo3290	porin	3.00E-106	Thauera sp. 63	Porin_4	0	Yes
azo3291	porin	1.00E-101	Thauera phenylacetica	Porin_4	0	Yes

Azo Number	Related Proteins	E value	Organism	Pfam Domains	TMH	Signal Peptide
azo3555	TonB-dependent receptor	0.00E+00	Thauera linaloolentis	Plug; TonB_dep_Rec	0	Yes
azo3556	TonB-dependent receptor	0.00E+00	Aromatoleum aromaticum	Plug; TonB_dep_Rec	0	Yes
azo3638	bifunctional enoyl-CoA hydratase/phosphate acetyltransferase	0.00E+00	Thauera linaloolentis	MaoC_dehydratas; PTA_PTB	0	No
azo3738	hypothetical protein	6.00E-97	Oxalobacter formigenes	Gcw_chp	0	Yes
azo3960	hypothetical protein	2.00E-161	Thauera sp. 63	YicC_N; DUF1732	0	No

FC: Fold change, given as BHΔtagF/BHΔtssM1tssK2

p-value according to 2-sided Student's t-test

Localization according to PsortB database

BLAST results considered hits with E value e-5 or lower

Pfam Domains according to Pfam.Sanger.Ac.UK

Transmembrane Helix (TMH) as predicted by CBS TMH Prediction Service

Signal Peptide as predicted by CBS Signal Peptide Prediction Service
